# Advances in Synchrotron Radiation‐Based Vacuum‐Ultraviolet Circular Dichroism for Biomolecular Structural Analysis

**DOI:** 10.1002/asia.202500996

**Published:** 2026-01-28

**Authors:** Koichi Matsuo, Satoshi Hashimoto, Ryota Imaura, Mohamed I. A. Ibrahim

**Affiliations:** ^1^ Research Institute for Synchrotron Radiation Science (HiSOR) Hiroshima University Higashi‐Hiroshima Japan; ^2^ Graduate School of Advanced Science and Engineering Hiroshima University Higashi‐Hiroshima Japan; ^3^ International Institute for Sustainability with Knotted Chiral Meta Matter (WPI‐SKCM^2^) Hiroshima University Higashi‐Hiroshima Japan; ^4^ Research Institute for Semiconductor Engineering Hiroshima University Higashi‐Hiroshima Japan

**Keywords:** biopolymers, proteins, saccharides, synchrotron radiation, vacuum‐ultraviolet circular dichroism

## Abstract

Vacuum‐ultraviolet circular dichroism (VUVCD) spectroscopy using synchrotron radiation is a powerful tool for characterizing the structures of biomolecules in aqueous solutions. When combined with bioinformatics, VUVCD enables detailed determination of protein secondary‐structure contents, segment numbers, and sequence distributions, while coupling with linear dichroism and shear flow techniques provides insights into the orientations of secondary structures. These analytical approaches have been applied to the structural characterization of membrane‐bound α‐synuclein (αS), a protein which transforms into amyloid fibrils associated with Parkinson's disease, successfully revealing the molecular mechanism of αS fibril‐formation on membranes. Furthermore, time‐resolved measurements using microfluidic device allow direct observation of protein structural dynamics during membrane interactions. The combination of VUVCD spectroscopy with molecular dynamics simulations in aqueous environments has also been applied to determine the absolute configurations of saccharides containing higher‐energy chromophores, thereby elucidating hydration structures involving intra‐ and intermolecular hydrogen bonds. Recent studies have further expanded these applications to monitor the conformational changes of extracellular polysaccharides and polyhydroxyalkanoates as functions of temperature and membrane interactions. These advancements highlight that integrating VUVCD spectroscopy with computational and other experimental approaches can significantly enhance the structural characterization of diverse biomolecules at molecular level.

## Graphical abstract

Vacuum‐ultraviolet circular dichroism spectroscopy using synchrotron radiation is operated at the Research Institute for Synchrotron Radiation Science (HiSOR), Hiroshima University. By combining the circular dichroism technique with bioinformatics, molecular dynamics, and linear dichroism, the spectral analysis methods have been advanced, enabling precise structural analyses of biomolecules in solution and elucidating their molecular functions.

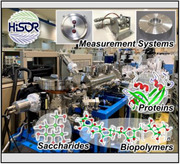

## 1 Introduction

Circular dichroism (CD) is a phenomenon in which left‐ and right‐handed circularly polarized light (CPL) are absorbed differently by optically active (chiral) molecules, reflecting their 3D steric structures with high sensitivity [1]. Therefore, CD spectroscopy is widely used to determine the absolute configurations of organic molecules, as well as to study the structures of biomolecules such as proteins, saccharides, and DNA [2]. Several CD spectroscopic techniques have been developed according to the wavelength region employed, including electronic CD [3], vibrational CD [4], and Raman optical activity [5]. Among them, electronic CD (hereafter abbreviated as CD) originates from electronic transitions (*n*–π* and π–π*) in the ultraviolet region, arising from absorption by chromophores such as carboxyl and amide groups within biomolecules.

Although CD does not provide atomic‐level structural information comparable to x‐ray crystallography or NMR, it is highly sensitive to conformational changes in biomolecules at the molecular level. CD measurements require only small sample volumes, independent of the molecular weight of the biomolecules and without the need for crystallization. Moreover, the measurements can be conducted under a wide range of solvent conditions that closely mimic the *in vivo* environment, including variations in temperature, pH, ionic strength, and the presence of additives such as biological membranes [6].

The use of synchrotron radiation (SR) as a light source has greatly enhanced the utility of CD spectroscopy, since CD spectra recorded using the SR light exhibit high signal‐to‐noise ratio and can be extended into the vacuum‐ultraviolet (VUV) region (wavelengths down to 120 nm) [7]. This spectral extension enables absorptions by high‐energy chromophores such as hydroxyl groups and acetal bonds (*n*–σ* and σ−σ*), [8] providing more detailed structural information that cannot be obtained by conventional CD instruments. At present, while some synchrotron facilities have been closed or relocated, SR‐based CD spectrometers are in operation at the Aarhus Storage Ring (Denmark), [9, 10]. Diamond Light Source (UK), [11, 12] the Research Institute for Synchrotron Radiation Science (HiSOR, Japan), and Synchrotron SOLEIL (France) [13, 14].

In Japan, a VUVCD spectrometer using an SR light source has been developed at HiSOR, Hiroshima University, since 1997 [15, 16]. This instrument has been extensively applied to the structural analysis of biomolecules in aqueous solutions. Recent developments, including integration with computational tools such as bioinformatics and molecular dynamics (MD) simulations, [17] as well as coupling with linear dichroism (LD), [18] and spatially and time‐resolved (TR) measurement systems, [19, 20] have further increased the potential and significance of SR‐based CD techniques. Further, research has extended the application of the technique to structural studies of biopolymers such as extracellular polysaccharides (EPS) [21, 22] and polyhydroxyalkanoates (PHAs), [23, 24] in the VUV region. These biopolymers exhibit structure‐dependent functional properties that depend strongly on their conformations in aqueous media. Owing to their chiral and flexible backbones, VUVCD enables precise characterization of their structures, crystalline, and intermolecular interactions.

In this paper, we provide an overview of the recent development and advancements of the VUVCD spectrometer utilizing an SR light source at HiSOR. We also introduce representative applications of this technique for the structural analysis of various biomolecules, together with progress of multiple international researches, including proteins, saccharides, and biopolymers such as EPSs and PHAs, along with a discussion of future perspectives.

## 2 VUVCD Spectrophotometer Using Synchrotron Radiation

HiSOR at Hiroshima University houses a compact SR facility equipped with a 700 MeV electron storage ring. The VUVCD spectrophotometer is installed on beamline 12 (BL‐12), [25] which covers a spectral range from 620 (2 eV) to 124 nm (10 eV). The light flux density at the sample position exceeds 1 × 10^12^ photons/s at 180 nm, enabling SR experiments to be performed without causing sample damaging in aqueous solutions. This section outlines the optical and measurement systems of the VUVCD spectrophotometer at HiSOR.

### 2.1 Optical System

**FIGURE 1:**
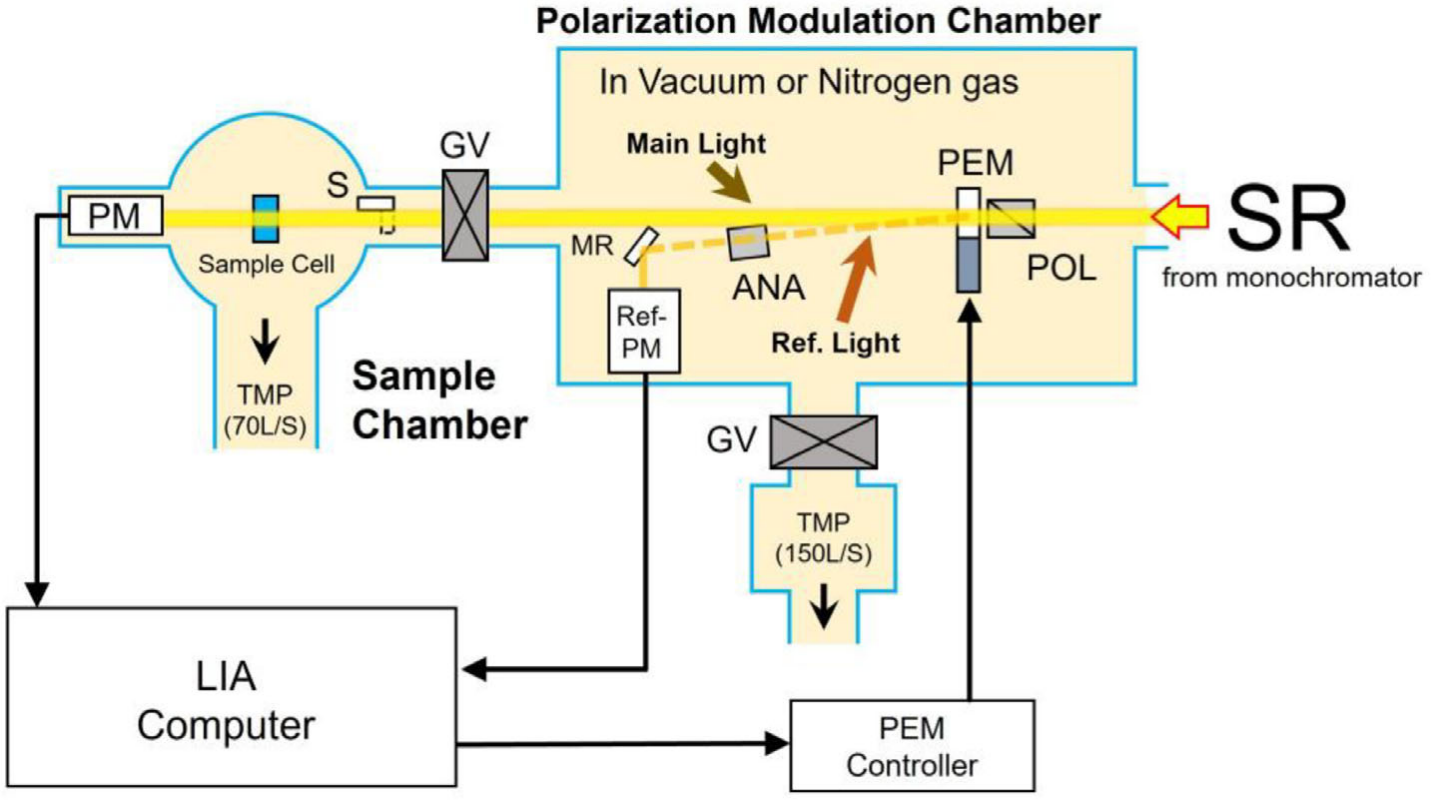
Schematic diagram of a vacuum‐ultraviolet circular dichroism spectrophotometer. (SR: Synchrotron radiation, POL: Linear polarizer, PEM: Photo‐elastic modulator, ANA: Analyzer, MR: Mirror, PM: Photomultiplier, GV: Gate valve, LIA: Lock‐in amplifier, TMP: Turbomolecular pump).

The optical system of the VUVCD instrument has been described in detail in previous studies [8, 26]. Briefly, as shown in Figure 1, monochromatic SR light is first divided into two orthogonal linear polarizations (main and reference beams) by an MgF_2_ Rochon‐type polarizer. These beams are then modulated into left‐ and right‐CPL at 50 kHz by a CaF_2_ photo‐elastic modulator. The main beam passes through the optical cell and is detected by a photomultiplier tube (Main‐PM). The signal (50 kHz) from the Main‐PM is synchronously rectified with the reference signal using a lock‐in amplifier (LIA) and recorded as a CD value. The reference beam is detected by another photomultiplier tube to provide the reference signal for the LIA. All optical elements are designed to operate under both a nitrogen atmosphere and high‐vacuum conditions (10^−4^–10^−5^ Pa), ensuring stable and high‐precision measurements in the VUV region down to 140 nm.

### 2.2 Measurement Systems

**FIGURE 2:**
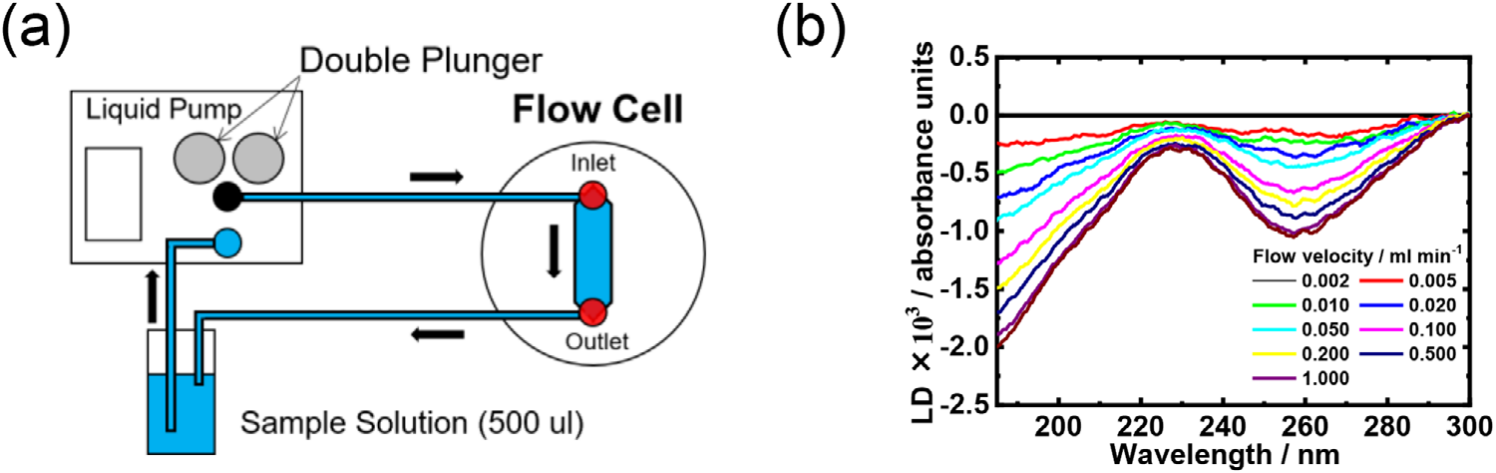
Flow cell for LD measurement. (a) Block diagram of the liquid‐circulation system with a dual‐plunger parallel‐flow pump and a flow cell for LD measurements. (b) LD spectra of calf thymus DNA from 300 to 185 nm at flow velocities ranging from 0.0002 to 1.0 mL/min. Adapted from Matsuo et al. (2016) [18].

Table 1 shows the photo, schematic diagram, and some specs of the representative cells used in the VUVCD instrument. The optical cell is typically used for VUVCD measurements of solution samples (protein, saccharide, DNA, etc.) at room temperature. The optical path length of this cell can be adjusted between 1.3 and 100 µm using thin Teflon spacers to minimize water absorption. Temperature‐controlled cell uses a donut‐type Peltier element to precisely control the solution temperature from −20°C to 100°C with an accuracy of ± 0.1°C. The SR light passes through the center of Peltier element and a dedicated optical cell. Since direct measurement of sample temperature is challenging, an auxiliary thermometer is placed at the sample position to calibrate the two sensors, enabling accurate estimation of the actual sample temperature. The flow cell is composed of inlet and outlet nanoports and two fused‐quartz windows. As shown in Figure 2a, when the nanoports were connected to a liquid pump, the sample solution can be circulated between the flow channel and pump, allowing long‐term measurements and in situ introduction of additives into the sample solutions during flow [18]. This circulation generates a high shear rate within the flow channel, orienting elongated molecules such as DNA or protofilaments and deforming globular liposomes into elliptical shapes [27]. The liquid circulation system can be applied to LD measurements to determine the orientation of chromophores and/or molecules within or bound to macromolecules (e.g., protofilaments, DNA, and biological membranes). Figure 2b shows the LD spectra of calf thymus DNA recorded at various flow velocities (0.0002–1.0 mL/min), indicating that the shear‐induced molecular orientation of DNA saturates at flow velocity of 1.0 mL/min.

**TABLE 1:** Typical cells used in VUVCD instrument.

Type	Optical cell	Temperature‐controlled cell	Flow cell for LD
Photo and schematic diagram	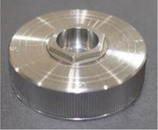	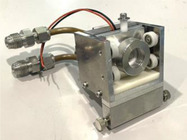	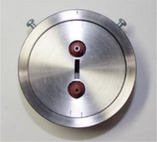
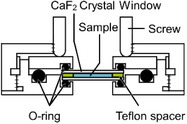	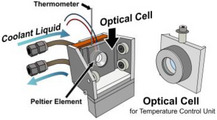	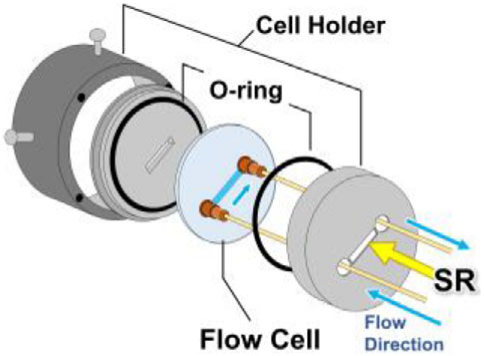
Feature	Standard measurements	Temperature‐dependent measurements	Flow‐ and angle‐dependent measurements
Path length	1.3–100 µm	1.3–100 µm	75µm
Temperature	Room temperature	−20°C to 100°C	5°C–50°C
Analysis	Structure analysis of biomolecules (protein, nucleic acid, and saccharide)	Thermal denaturation of biomolecules and phase transitions of membranes	Molecular orientation analysis of proteins and nucleic acids

These dedicated measurement systems enable high‐precision VUVCD and LD measurements of biomolecules such as proteins, DNA, and polysaccharides under a variety of experimental conditions.

## 3 Structural Analysis of Biomolecules

### 3.1 Proteins

The structural changes of proteins are critical for the expression of their biological phenomena and functions. Accordingly, the structural change processes including protein denaturation and folding, which are influenced by environmental factors such as pH and temperature, have long been major research topics in protein science [28, 29]. This structural change can also be induced by interactions with other biological components such as cell membranes. Such interactions are of particular social and medical importance because they are linked to diverse biological phenomena, including pathogenesis of neurological diseases (e.g., Alzheimer's disease), [30] antibacterial functions against drug‐resistant bacteria, [31, 32] and transport of drugs into cells [33, 34, 35]. Therefore, understanding how proteins interact with membranes, and how these interactions contribute to biological processes, is not only of academic significance, but also of highly relevant to medicine and industry.

Membrane‐bound proteins, which are water‐soluble proteins in their native state but adopt altered conformations upon membrane interaction, remain difficult targets to analyze using conventional spectroscopic methods such as x‐ray crystallography and nuclear magnetic resonance (NMR). This difficulty arises because membranes often hinder crystallization, complicate sample preparation, and make spectral interpretation more challenging. Even state‐of‐the‐art computational approaches such as AlphaFold2 [36] and AlphaFold3 [37] still face limitations in accurately predicting membrane‐induced conformational changes.

Protein CD spectroscopy can detect structural motifs associated with peptide bond conformations, whose dihedral angles depend strongly on secondary structure, such as α‐helices and β‐strands. CD spectrophotometer using SR light source has been successfully applied to the structural studies of membrane‐bound proteins involved in neurological diseases, antibacterial diseases, and transport functions [17, 38, 39, 40]. Myelin basic protein (MBP), which is a representative membrane‐bound protein, plays an important role in the formation and stabilization of multilayered myelin in the central nervous system [41, 42]. Kursula et al. employed SR‐based CD and oriented CD (OCD) spectroscopy to elucidate the structural properties and membrane‐associated conformations of MBP [43, 44, 45]. CD measurements demonstrated that MBP formed a predominantly disordered conformation in aqueous solution but partially formed α‐helical structures upon interaction with lipid bilayers. Furthermore, OCD measurements revealed that these helical segments adopted specific orientations with respect to the membrane surface, being aligned nearly parallel or at a defined tilt angle relative to the lipid bilayer. In particular, membranes containing negatively charged lipids were shown to promote both structural ordering and orientational alignment of MBP through electrostatic interactions. Similarly, the combined use of CD and OCD spectroscopy has proven useful for the structural analysis of the membrane‐bound state of the antimicrobial peptide magainin 2 (M2) [46, 47]. CD measurements showed that M2 adopted a predominantly disordered conformation in aqueous solution but formed an α‐helical structure in the presence of membranes. OCD analyses further revealed that the α‐helical segment of M2 was oriented parallel to the membrane surface at low peptide‐to‐lipid ratios, whereas at higher ratios it adopted a tilted or partially inserted orientation. These studies demonstrate that the combined use of CD and OCD provides a powerful approach for characterizing partial folding and orientational behavior in membrane‐associated disordered proteins. Thus, the integration of CD with other spectroscopic methods is highly effective. More recently, a TR apparatus has been developed and integrated into the VUVCD instrument, enabling the determination of kinetic parameters involved in membrane interactions, such as association rate constants and the identification of transient protein intermediates [20].

In this section, we highlight studies on membrane‐protein interactions investigated using VUVCD spectroscopy in combination with other experimental and theoretical techniques. Particular emphasis is placed on the membrane‐associated amyloid fibril formation of α‐synuclein (αS) in relation to Parkinson's disease (PD), [17, 40] and on the dynamic observation of the membrane‐protein interaction processes using TR‐VUVCD apparatus [20].

#### 3.1.1 Structure and Function Studies of Parkinson's Disease‐Causing αS

PD is a neurodegenerative disorder associated with amyloidosis and is characterized by the accumulation of amyloid fibrillar aggregates, known as Lewy bodies, in neurons. The aggregation process is strongly linked to interactions between αS and synaptic vesicle membranes [30, 48, 49]. αS is a 140‐amino acid residue comprising three distinct domains: the N‐terminal domain (residues 1–60), the non‐amyloid β‐component (NAC) domain (residues 61–95), and the C‐terminal domain (residues 96–140). Cellular salts such as NaCl and CaCl_2_ play an important role in modulating the characteristics of αS amyloid fibrils, because these ions directly influence the electrostatic interactions between αS molecules and between αS and membranes, while perturbing hydrophobic interactions between αS and membrane at the membrane interface [50, 51, 52, 53]. Consequently, the impacts of salt type and concentration on both membrane interaction and fibril formation mechanisms of αS have been widely investigated [54, 55, 56, 57, 58, 59, 60]. In this study, VUVCD and LD spectroscopies [38] were used to probe the structural features of αS bound to membranes in the presence and absence of salt (i.e., NaCl). Based on these experimental results, MD simulations were performed to resolve the salt effects on αS‐membrane interactions at both the secondary‐structure and amino‐acid–residue levels.

##### 3.1.1.1 Mechanism of Fibril Formation by αS Bound to Synaptic Mimic Membranes

To investigate the role of salt in αS amyloidogenesis, model membranes mimicking the lipid composition and size of synaptic vesicles were prepared. Fibrillation kinetics were monitored at varying lipid‐to‐protein (L/P) molar ratios using thioflavin T (ThT) fluorescence. As a result, the amyloid fibrils were observed only under low (∼ 20) L/P ratios in the presence of NaCl, whereas no aggregation was detected under salt‐free conditions or at high L/P ratios, even in the presence of salt [61].

**FIGURE 3:**
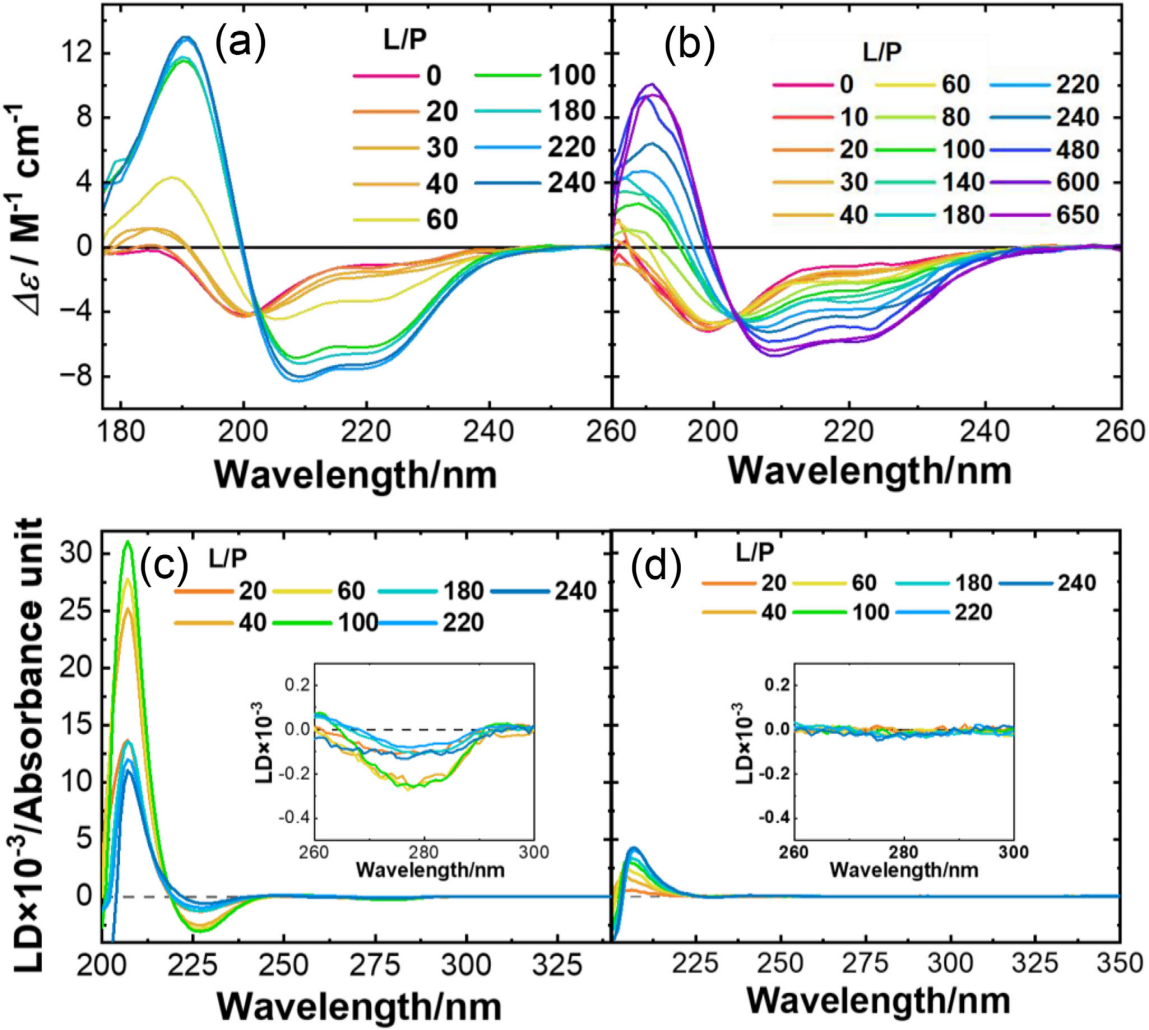
VUVCD and LD spectra of αS at various L/P ratios. VUVCD spectra in the (a) absence and (b) presence of NaCl (0.1 M). LD spectra in the (c) absence and (d) presence of NaCl (0.1 M). The αS concentration was fixed at 50 µM for all CD and LD measurements. Insets in (c) and (d) show magnified images of the 275 nm region. Adapted from Imaura et al. (2024) [17].

As αS fibrillation originates from its membrane‐bound state, the membrane‐bound structure of αS was further analyzed at the molecular (i.e., secondary structure) level under salt‐present and salt‐absent conditions at various L/P ratios using VUVCD spectroscopy (Figure 3a,b). The spectra revealed a random coil to α‐helix transition with increasing L/P ratios, regardless of the salt conditions, but the α‐helical content was consistently lower in the presence of NaCl. This reduction in α‐helicity is suggested to contribute to the fibril formation. To further investigate the membrane binding and orientation of the α‐helical structures and tyrosine (Tyr: Y) residues in membrane‐bound αS, LD measurements were performed under the same conditions as the VUVCD experiments (Figure 3c,d). The results revealed that, in both salt‐free and salt‐containing environments, α‐helical segments were aligned parallel to the membrane surface, as inferred from the LD signs. However, the fraction of membrane‐bound helices decreased in the presence of NaCl, as indicated by reduced LD intensity. Additionally, analysis of Y residues revealed a similar salt effect: in the absence of NaCl, Tyr aromatic rings were oriented toward the membrane surface, whereas this interaction was disrupted in the presence of salt. These findings suggested that the membrane‐bound regions of αS (helix regions and Tyr residues) observed under salt‐free conditions were partially exposed to solvent in the presence of salt, contributing to fibril formation.

To elucidate the behavior of membrane‐bound Tyr residues in αS (Y39 in the N‐terminal domain, and Y125, Y133, and Y136 in the C‐terminal domain), MD simulations were performed for both terminal domains in the presence of membranes. The simulations revealed that the N‐terminal domain consistently formed α‐helix and bound to the membrane regardless of salt conditions. In contrast, the C‐terminal domain remained in a random coil structure and only weakly interacted with the membrane. Under salt‐present conditions, some hydrophobic residues in the C‐terminal domain, including Y125, no longer engaged in membrane interactions and became solvent‐exposed, suggesting that this hydrophobic region induced fibril formation through intermolecular interactions with other αS molecules. The relative proportions of free and membrane‐bound αS were calculated from VUVCD data, revealing that at low L/P ratios, a substantially higher proportion of free αS was present compared to high L/P ratios. The abundance of free αS could largely promote intermolecular interactions with membrane‐bound αS, contributing to fibril formation.

**FIGURE 4:**
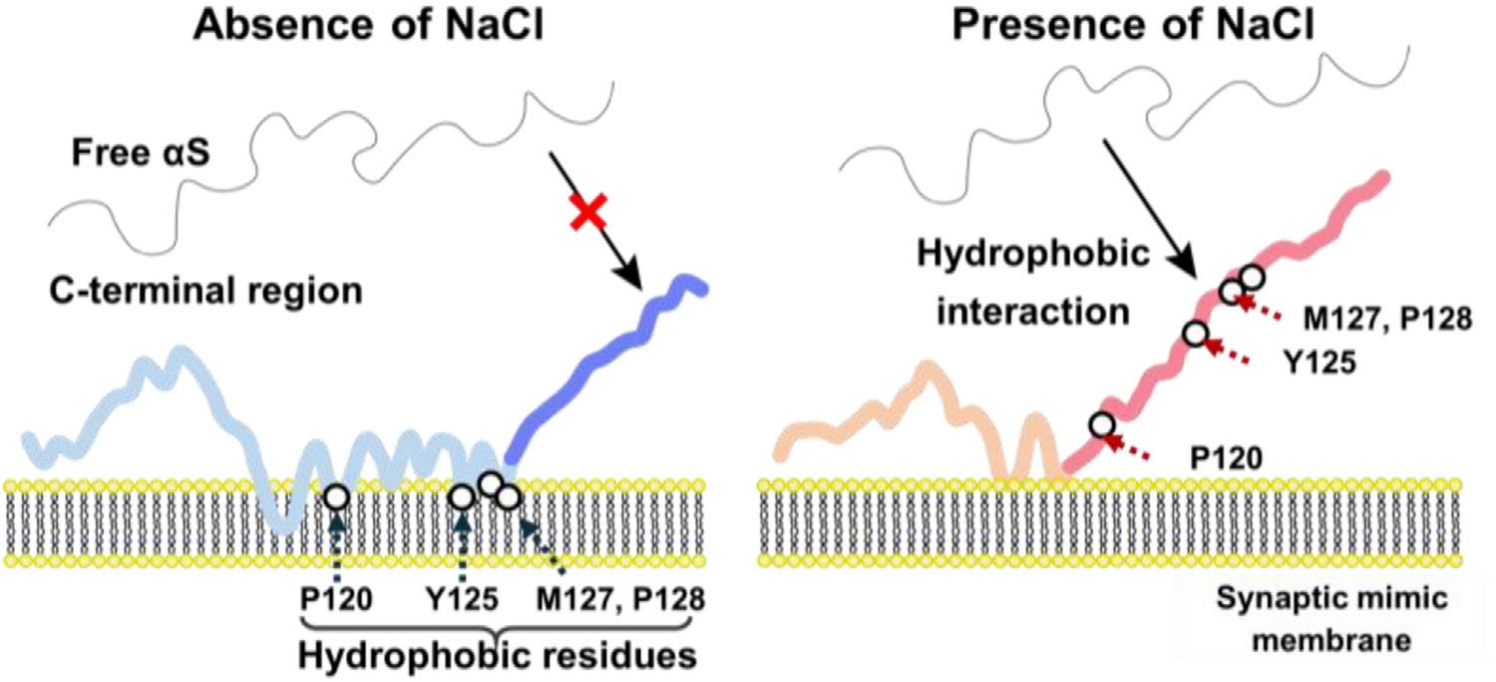
Proposed mechanism of αS fibril formation. The C‐terminal domain of αS remains flexible even after the N‐terminal domain interacts with membrane, regardless of NaCl. In the absence of NaCl, hydrophobic residues in the C‐terminal domain tightly interact with the membrane, preventing free αS from accessing membrane‐bound αS. In contrast, in the presence of NaCl, these hydrophobic residues become solvent‐exposed, allowing free αS to interact with membrane‐bound αS and promoting intermolecular association that drive amyloid fibril formation. Adapted from Imaura et al. (2024) [17].

Based on these results, the fibril formation mechanism was speculated as illustrated in Figure 4. Initially, the N‐terminal domain would interact with the membrane surface, facilitating the accumulation of αS on the membrane, while the C‐terminal domain remains flexible. In the absence of NaCl, hydrophobic residues in the C‐terminal domain tightly interact with the membrane, preventing free αS from accessing membrane‐bound αS. In contrast, in the presence of NaCl, these hydrophobic residues become exposed to the solvent, allowing free αS to interact with membrane‐bound αS. These interactions promote the intermolecular association that drive amyloid fibril formation.

These findings suggested that solvent‐exposed hydrophobic residues, including Tyr residues in the C‐terminal domain, facilitated hydrophobic interactions with free αS, thereby accelerating amyloid fibril formation.

##### 3.1.1.2 Study of the Fibril Formation Mechanism With Structural Polymorphism of the NAC Domain Bound to Anionic Membrane

**FIGURE 5:**
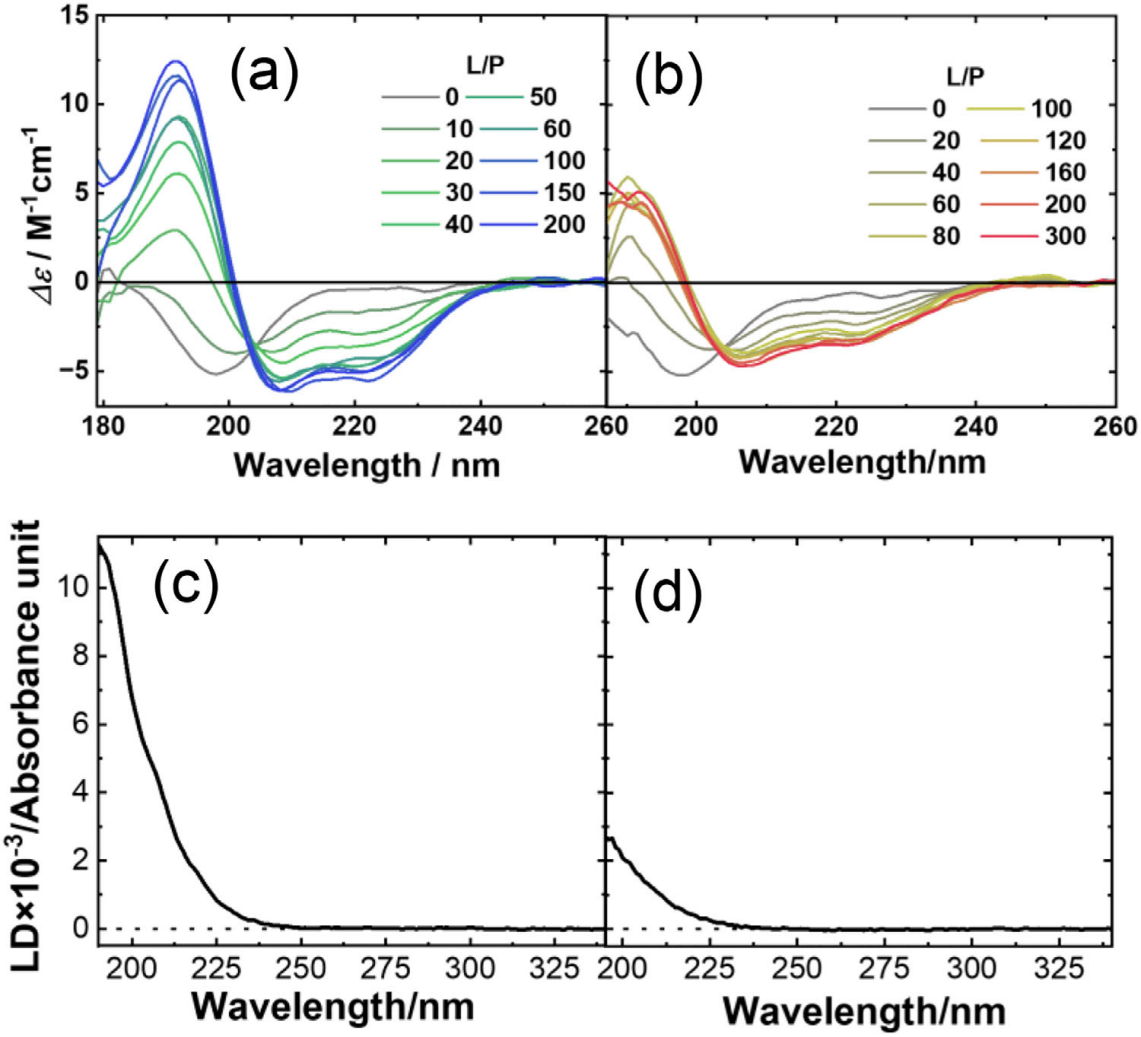
VUVCD spectra of αS_57–102_ at various L/P ratios in the (a) absence and (b) presence of NaCl (0.1 M). LD spectra at L/P = 200 in the (c) absence and (d) presence of NaCl (0.1 M). The αS_57–102_ concentration was fixed at 50 µM for all CD and LD measurements. Adapted from Imaura et al. (2025) [40].

The NAC domain of αS is critical for fibrillation, because intermolecular interactions between NAC domains promote β‐sheet formation and play a key role in nucleation [62, 63, 64, 65, 66]. Amyloid fibrils of the NAC domain‐containing peptide αS_57–102_ were examined using ThT fluorescence measurements and transmission electron microscopy. The results showed that thin fibrils formed only at low L/P ratios in the absence of salt, whereas two distinct fibril morphologies (thin and thick) were observed at low L/P ratios, and only thick fibrils were detected at high L/P ratios in the presence of salt. To investigate the origin of this structural polymorphism, VUVCD spectra of membrane‐bound αS_57–102_ were measured in the presence and absence of salt, as shown in Figure 5a,b. Analysis of the CD spectra for the membrane‐bound states (*L*/*P* = 200, with and without salt) revealed that αS_57–102_ transitioned from a random coil to an α‐helix structure upon interaction with membranes in both conditions, but the α‐helical content was significantly reduced in the presence of salt.

**FIGURE 6:**
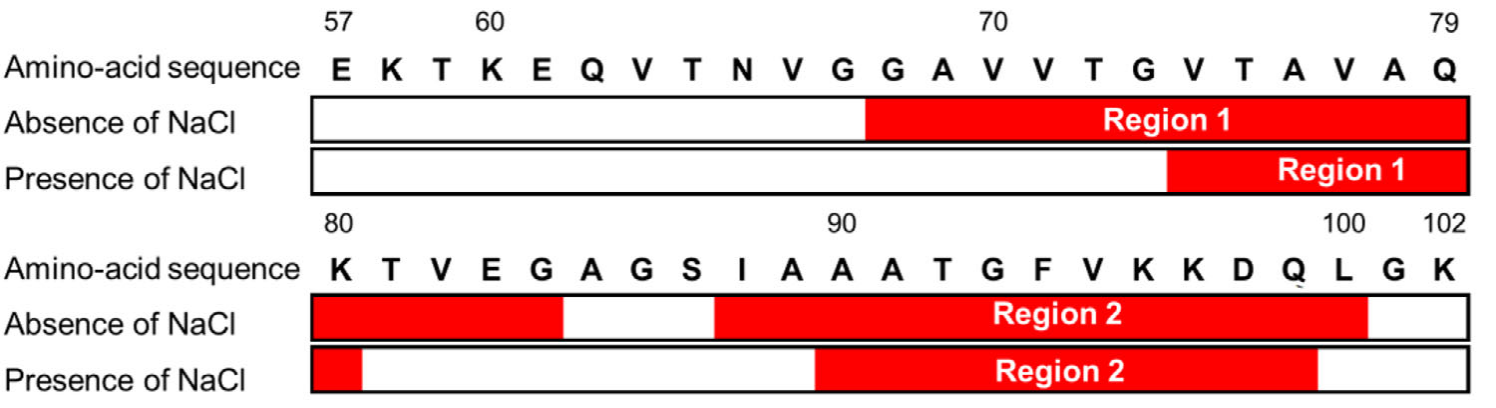
Sequences of secondary structures of membrane‐bound αS_57–102_ in the absence and presence of NaCl (0.1 M). Red regions represent α‐helix segments. Adapted from Imaura et al. (2025) [40].

Figure 6 shows the secondary‐structure sequences of membrane‐bound αS_57–102_, predicted using the VUVCD‐NN method [67]. The results revealed the formation of two helical regions (regions 1 and 2) on the membrane, irrespective of salt conditions. However, in the presence of salt, region 1 displayed a substantial shortening of its helical length, suggesting that this reduction in α‐helicity may contribute to the observed fibril polymorphism.

**FIGURE 7:**
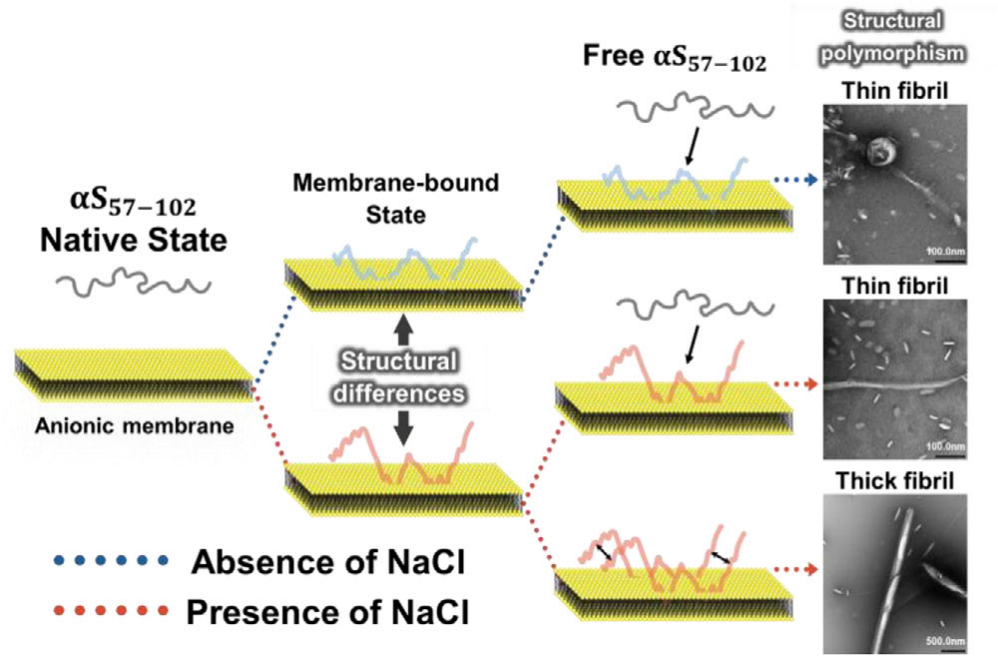
Proposed fibril formation pathways depending on the L/P ratio. At low L/P ratios, in both the presence and absence of salt, interactions between free and membrane‐bound αS_57–102_ predominantly drove fibril nucleation. At high L/P ratios, where membrane‐bound αS_57–102_ dominates, nucleation occurs by interactions between membrane‐bound αS_57–102_ via exposed hydrophobic amino acids. Adapted from Imaura et al. (2025) [40].

LD spectroscopy further showed that these helices were aligned parallel to the membrane surface, regardless of salt presence (Figure 5c,d). To further characterize the membrane interaction sites of αS_57–102_, MD simulations of this domain under both conditions were conducted. The simulations identified two regions interacting with the membrane interior, and the locations and orientations of the membrane‐bound regions, in the presence and absence of salt, were consistent with the VUVCD and LD results. Furthermore, the regions that interacted with the membrane under salt‐free conditions but became solvent‐exposed in the presence of salt were enriched in hydrophobic amino acids, suggesting that this hydrophobic region play a critical role in fibril formation through intermolecular interactions. The proportions of free and membrane‐bound αS_57–102_ ratios were estimated from VUVCD data. As a result, under salt‐present conditions, two distinct fibril formation pathways were identified depending on the L/P ratio as described in Figure 7. At low L/P ratios, in presence or absence of salt, where the proportion of membrane‐bound αS_57–102_ was lower, fibril nucleation was driven primarily by interactions between free and membrane‐bound αS_57–102_. In contrast, at high L/P ratios, where membrane‐bound αS_57–102_ predominated, nucleation occurred through interactions among membrane‐bound αS_57–102_ via exposed hydrophobic amino acids. These results demonstrated that salt‐induced exposure of hydrophobic regions in αS_57–102_ contributed to the fibril formation through two distinct intermolecular interactions: between free and membrane‐bound αS_57–102_, or between membrane‐bound αS_57–102_, depending on the L/P ratio. These mechanisms have led to structural polymorphisms that varied in fibril thickness.

### 3.2 Dynamic Observation of the Membrane Interaction Processes of β‐Lactoglobulin by Time‐Resolved Apparatus

The VUVCD spectroscopy has been applied to the structure analysis of several membrane‐bound proteins; however, most studies have been limited to static conditions. Information on structural dynamics during membrane interaction processes is essential for elucidating mechanism of protein‐membrane interactions and the expression mechanism of biological function on the membrane at molecular level. In this study, to observe the structural dynamics of proteins during the membrane interaction, from native (N‐) to membrane‐bound (M‐) states in real time, we developed a TR apparatus and integrated it with VUVCD spectrophotometer. Using this system, membrane‐induced structural changes of β‐lactoglobulin (bLG) were analyzed using model membrane, lysoDMPG micelles.

**FIGURE 8:**
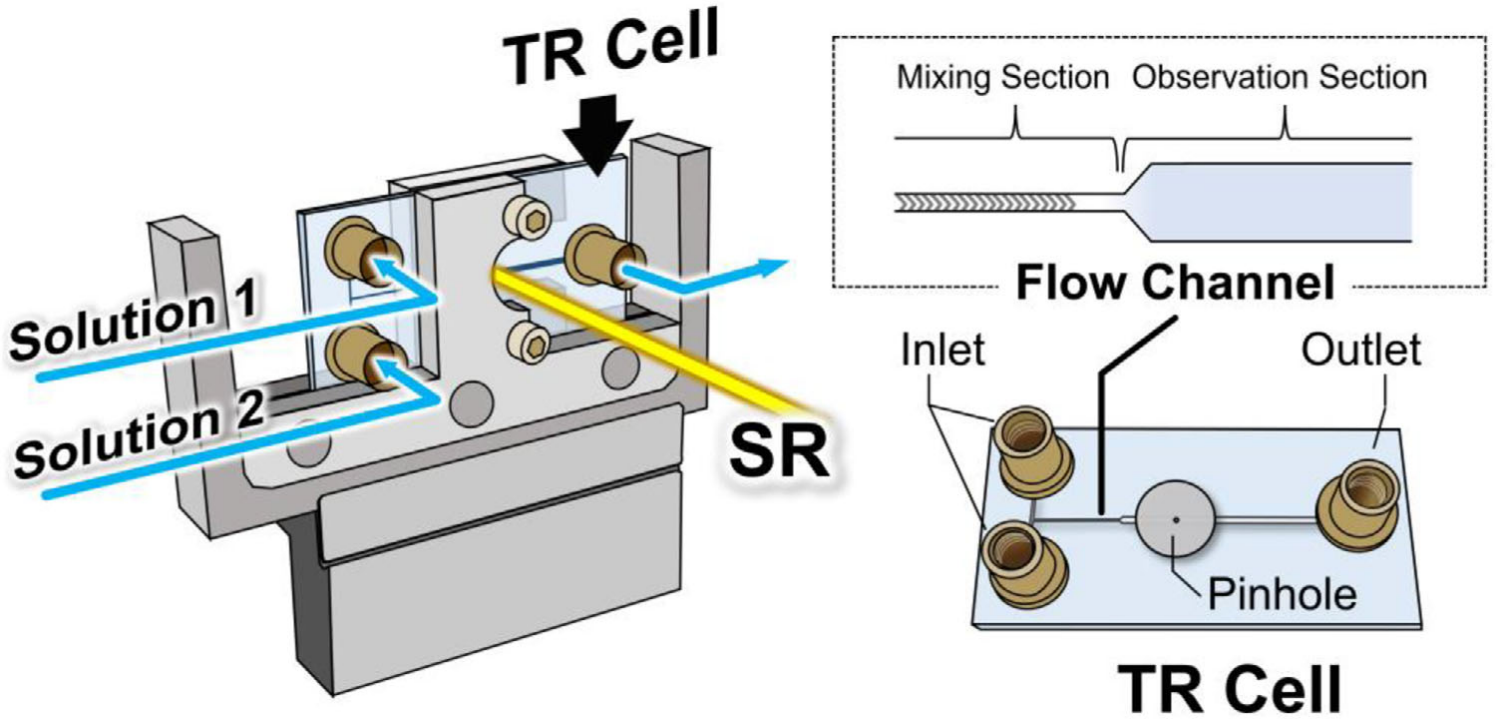
Schematic illustration of the microfluidic TR cell. Two solutions (protein and membrane) are injected from separate inlets and mixed in the herringbone‐grooved mixing section of the flow channel before reaching the observation section, where the SR beam passes through. The TR cell consists of two inlets, one outlet, and a pinhole.

A microfluidic TR cell made from synthetic quartz had two inlets, one outlet, and a T‐shaped microchannel as shown in Figure 8. The channel is divided into two sections, a mixing section and an observation section. The herringbone groove pattern in the mixing section was designed to efficiently and quickly mix two different solutions from the two inlets. Protein and membrane solutions were injected separately from each inlet and then reach the measurement section in which the SR passes. The reaction time was controlled by adjusting the flow rate of the syringe pump.

**FIGURE 9:**
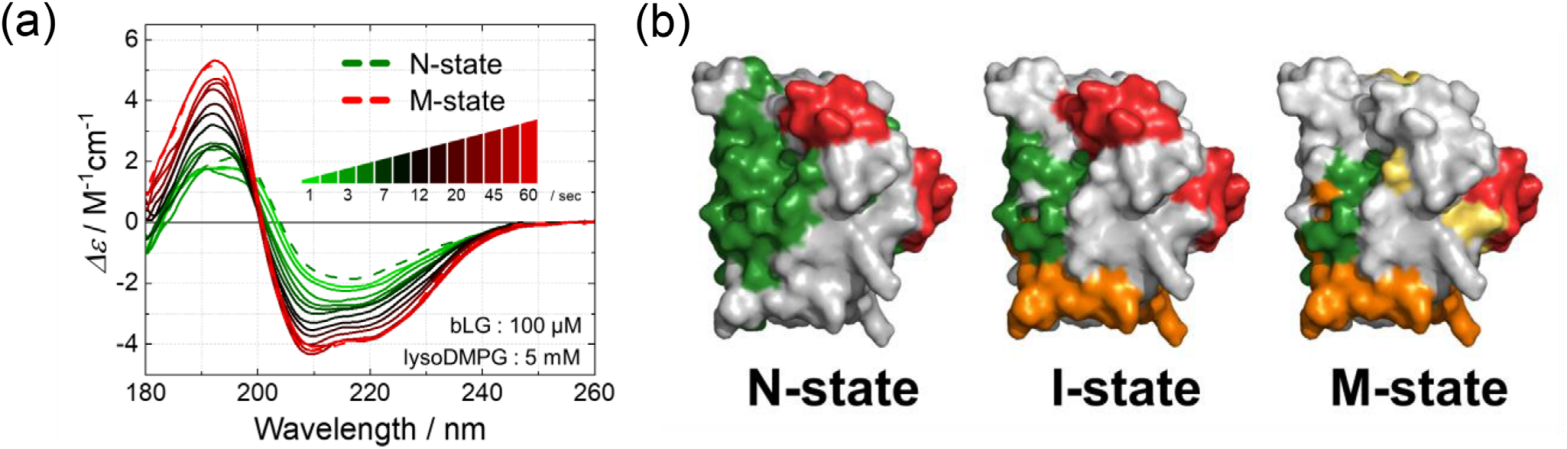
(a) TR‐VUVCD spectra of β‐lactoglobulin (bLG, 100 µM) interacting with lysoDMPG micelles (5 mM), showing structural changes from the N‐state (green) to the M‐state (red) within 1–60 s. (b) Mapping of secondary structure changes onto the 3D structure (PDB ID: 1BEB). In the N‐state, α‐helices, β‐strands, and other structures are shown in red, green, and white, respectively. Newly formed solvent‐exposed α‐helices in the I‐state are indicated in orange, and additional hydrophobic α‐helices in the M‐state are shown in yellow. Adapted from Hashimoto and Matsuo (2024) [20].

The structural changes of bLG induced by interaction with lysoDMPG phospholipid molecules were monitored using a TR apparatus over a reaction time range of 1–60 s as shown in Figure 9a. To obtain the kinetic parameters, global fitting analysis of the TR data set was performed, which revealed two association rate constants, suggesting the presence of an intermediate (I‐) state during the transition process from N‐ to M‐states. The secondary structures, including contents, numbers of segments, and sequences, of bLG in the N‐, I‐, and M‐states were analyzed using the SELCON3 and VUVCD‐NN methods [67, 68]. To visualize the structural changes, the secondary structures of each state were mapped onto the 3D structure of native bLG (1BEB), as shown in Figure 9b. As a result, in the I‐state, two newly formed helical regions (orange) were located in solvent‐exposed areas and carried positive net charges. In the M‐state, three newly formed helical regions (yellow) were mainly located within the inner barrel of bLG and were enriched in hydrophobic residues. Given that the lysoDMPG micelle surface is negatively charged and its interior is highly hydrophobic, these results indicated that the N‐ to I‐state and I‐ to M‐state transitions are primarily driven by electrostatic and hydrophobic interactions with membrane, respectively. Overall, these findings demonstrated the capability of the TR‐VUVCD system as a robust tool for characterizing protein‐membrane interactions at the molecular level.

In this study, left‐ and right‐CPL were generated using a combination of POL and PEM (Figure 1). In parallel, an ultrafast time‐resolved CD measurement system exploiting the intrinsic circular polarization of SR has been developed at Synchrotron SOLEIL [69]. Because membrane‐induced proteins structural changes can occur on very fast timescales depending on the membrane compositions, the usage of such high‐speed CD instrumentation will be valuable for further elucidating the mechanisms of protein‐membrane interactions.

### 3.3 Saccharides

Various classes of saccharides are present in living organisms, where they serve crucial functions in various biological processes. These include energy storage (e.g., glycogen and starch), the formation of structural components such as plant cell walls and arthropod exoskeletons (e.g., cellulose and chitin), protein–cell membrane recognition events (e.g., mannose and sialic acid), and protection against environmental stresses such as cold and desiccation (e.g., trehalose) [70, 71, 72, 73]. In addition, saccharides exhibit characteristic physical properties, including gelation (e.g., carrageenan) and retrogradation (e.g., amylose) [74, 75, 76].

Similar to proteins, CD spectroscopy is a powerful tool for probing the structures of saccharides in solution. This technique can tolerate a wide range of sugar complexities, including extensive branching, high viscosity, and large molecular weight ranges, and can be performed under diverse solvent conditions such as varying pH, temperature, and salt concentrations.

Saccharide CD is sensitive to chromophores arising from functional groups such as acetamido, carboxyl, acetal, and hydroxyl groups, and its CD spectra can be roughly divided into three wavelength regions. The two most common substituents (acetamido and carboxyl groups) display CD bands associated with the *n*–π* transitions at 200–240 nm and the π–π* transitions at 180–200 nm in the far‐ultraviolet (far‐UV) region. While the *n*–σ* transitions of acetal and hydroxy groups of unsubstituted saccharides produce CD bands at 140–180 nm in the VUV region. Thus, VUVCD measurements using an SR source are especially effective for determining the configurations of acetal linkages and hydroxyl groups, which form the backbone of all saccharides. These backbone configurations strongly influence both the physical properties and biological functions of saccharides, via intra‐ and intermolecular hydrogen bonding, as well as hydration interactions with water [77]. For instance, differences in hydroxyl group orientations between glucose and mannose alter carbohydrate‐lectin binding constants, [78, 79] while equatorial hydroxyl groups contribute to protein stabilization [80].

The CD spectroscopy has also been applied to the structural analysis of glycosaminoglycans (GAGs) or substituted saccharides due to its high sensitivity to chromophores such as acetamido and carboxyl groups [81, 82, 83, 84, 85]. Early studies measured CD spectra of various GAGs, including hyaluronic acid and chondroitin sulfates, in aqueous solutions, revealing characteristic amide and carboxyl transitions above 185 nm. Subsequent studies extended CD measurements into the VUV region (down to 170–175 nm) for several GAGs using SR light source, enabling more detailed structural characterization. The SR‐based CD technique was further applied to adenine‐containing nucleosides and nucleotides with ribose and deoxyribose, where the spectra were sensitive to molecular structure and adenine protonation. In these systems, CD signals mainly arose from *n*–π* transitions of adenine and weaker *n*–σ* transitions of saccharides, enabling discrimination among different saccharide conformations [86]. Thus, although CD is highly sensitive to the structures of substituted saccharides, this section highlights recent progress in the structural characterization of unsubstituted saccharides, containing only acetal and hydroxyl groups, using VUVCD spectroscopy [87, 88].

### 3.4 Monosaccharide CD Spectra

**FIGURE 10:**
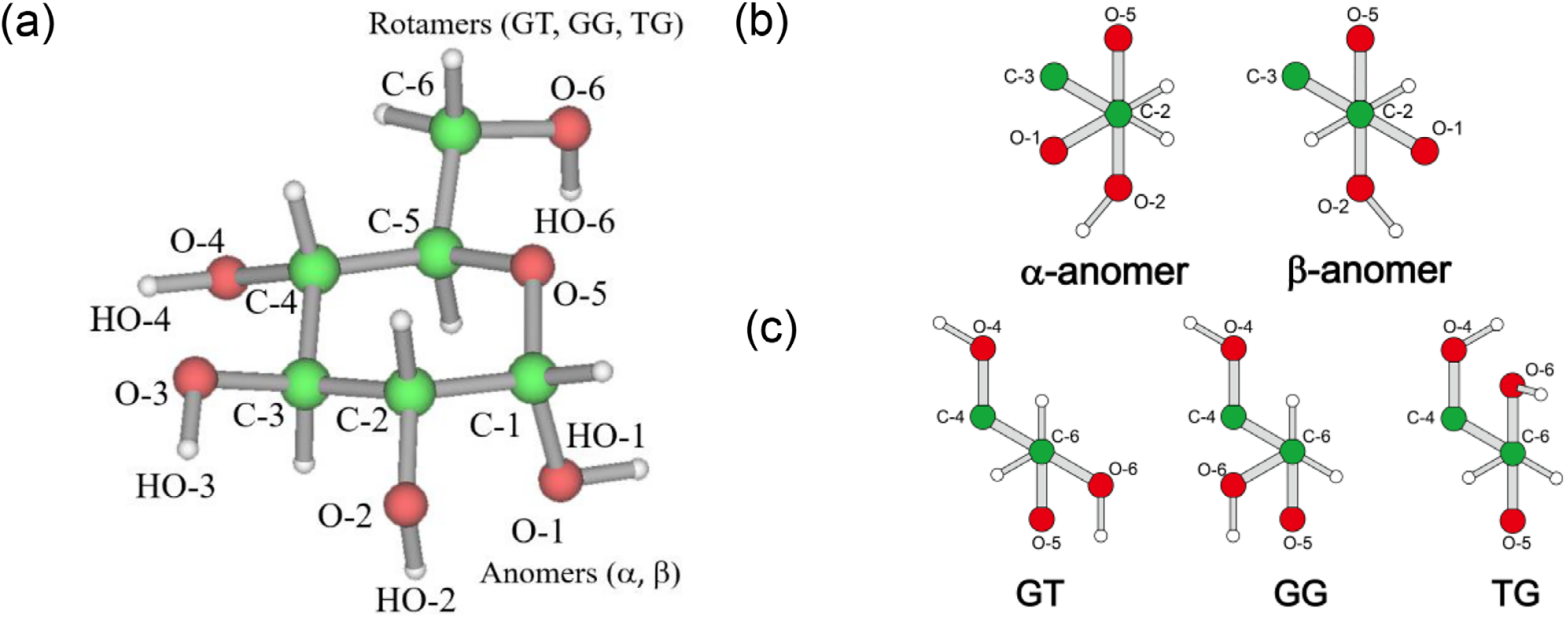
(a) Chemical structure of d‐glucose. (b) Two anomeric configurations (α and β) of the hydroxyl group at C‐1. (c) Gauche–gauche (GG), gauche–trans, (GT), and trans–gauche (TG) rotamers of hydroxymethyl group at C‐5. Adapted from Matsuo et al. (2020) [87].

Monosaccharides in aqueous solution exist as a complex equilibrium of multiple conformations. Figure 10 illustrates the chemical structure of d‐glucose (d‐Glc), which exhibits two anomeric configurations (α and β) depending on the orientation of the hydroxyl group at C‐1 (Figure 10b), three rotamers of the hydroxymethyl group at C‐5 (Figure 10c), and two chair conformations (^4^C_1_ and ^1^C_4_). The gauche–gauche (GG), gauche–trans (GT), and trans–gauche (TG) rotamers differ in the positions of the hydroxyl oxygen at C‐6 with respect to the ring oxygen. Since saccharides preferentially adopt the ^4^C_1_ pyranose conformation, CD spectroscopic studies typically consider six principal conformers: α‐GG, α‐GT, α‐TG, β‐GG, β‐GT, and β‐TG.

**FIGURE 11:**
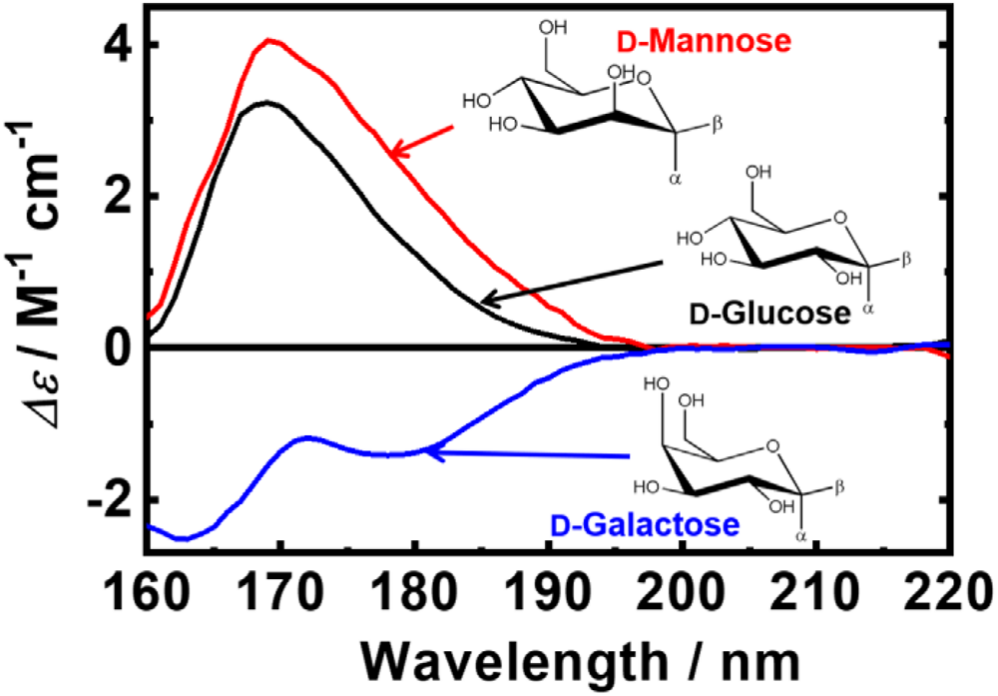
Experimentally observed VUVCD spectra of d‐glucose (black, concentration 10%(w/v)), d‐mannose (red, concentration 5%(w/v)), and d‐galactose (blue, concentration 5%(w/v)) in aqueous solution. Adapted from Matsuo and Gekko (2004) [76].

The VUVCD spectra of three representative monosaccharides in aqueous solution (d‐Glc, d‐mannose, and d‐galactose), are shown in Figure 11 [89]. Despite their structural similarities, these monosaccharides exhibit remarkably different spectral features. For instance, d‐Glc and d‐galactose differ only at the C‐4 position, where glucose has an equatorial hydroxyl group while galactose has an axial one (Figure 11). Nevertheless, glucose displays a positive CD peak near 170 nm, whereas galactose shows two negative peaks around 165 and 180 nm, highlighting the high sensitivity of VUVCD to hydroxyl group orientation. The CD bands observed in the 160–180 nm range are predominantly assigned to *n*–σ* electronic transitions of the ring oxygen, [90] which are strongly influenced by the neighboring hydroxyl group at C‐1 and the hydroxymethyl group at C‐5.

### 3.5 Theoretical Analysis of Monosaccharide CD Spectra

**FIGURE 12:**
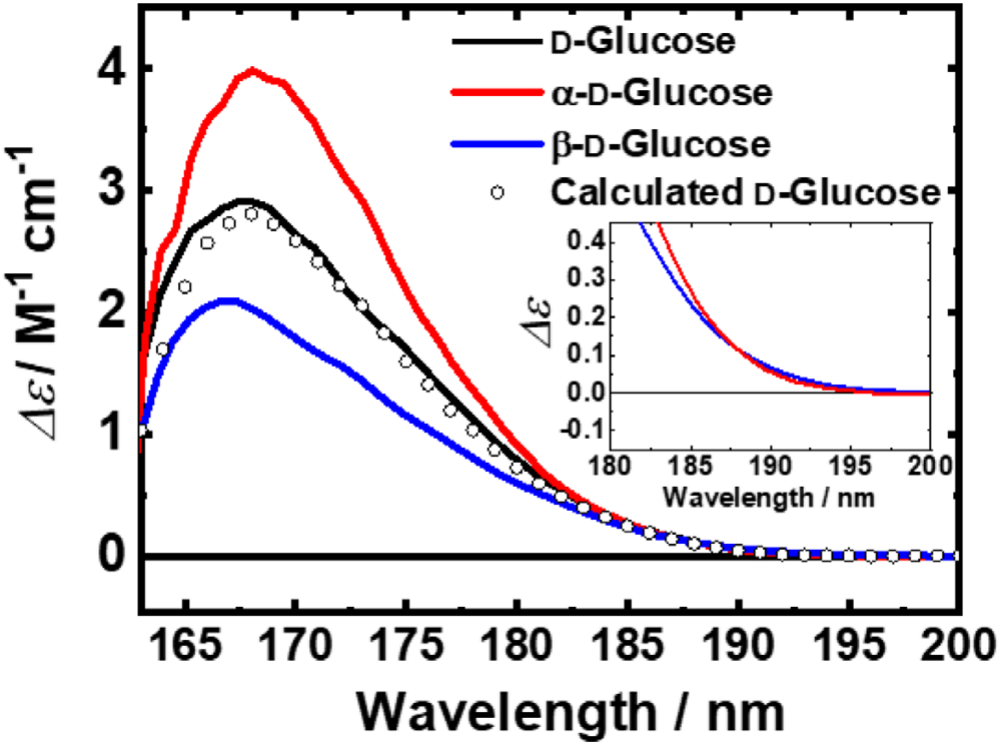
Experimentally observed VUVCD spectra of d‐Glucose, α‐d‐Glucose, and β‐d‐Glucose in aqueous solution. The calculated spectrum of d‐Glucose was obtained as a linear combination of the α‐d‐Glucose and β‐d‐Glucose spectra, weighed by their population ratio. The inset shows an enlarged view of the spectra of α‐d‐Glucose and β‐d‐Glucose from 200 to 180 nm. Adapted from Matsuo et al. (2020) [87].

Estimating the individual CD contributions of the C‐5 rotamer conformations is challenging because these conformers coexist in equilibrium in solution. Figure 12 presents the VUVCD spectra of d‐Glc, α‐d‐glucose (α‐d‐Glc), and β‐d‐glucose (β‐d‐Glc). All three spectra display a common positive peak near 168 nm; however, this peak is slightly blue‐shifted and showed decreasing intensity in the order: α‐d‐Glc > d‐Glc > β‐d‐Glc. Additionally, the spectra of α‐d‐Glc and β‐dGlc intersected at ∼185 nm, as highlighted in the inset.

The d‐Glc spectrum shown in Figure 12 was estimated as a linear combination of the α‐ and β‐anomer spectra, weighted by their population ratio [91]. The close agreement between the calculated and experimental spectra demonstrated that the CD contributions of the d‐Glc anomers are additive. Hence, the individual CD spectra of the six d‐Glc conformers (α‐GT, α‐GG, α‐TG, β‐GT, β‐GG, and β‐TG) were calculated using MD simulations combined with time‐dependent density functional theory (TDDFT), and the calculated results were compared with experimental data.

**FIGURE 13:**
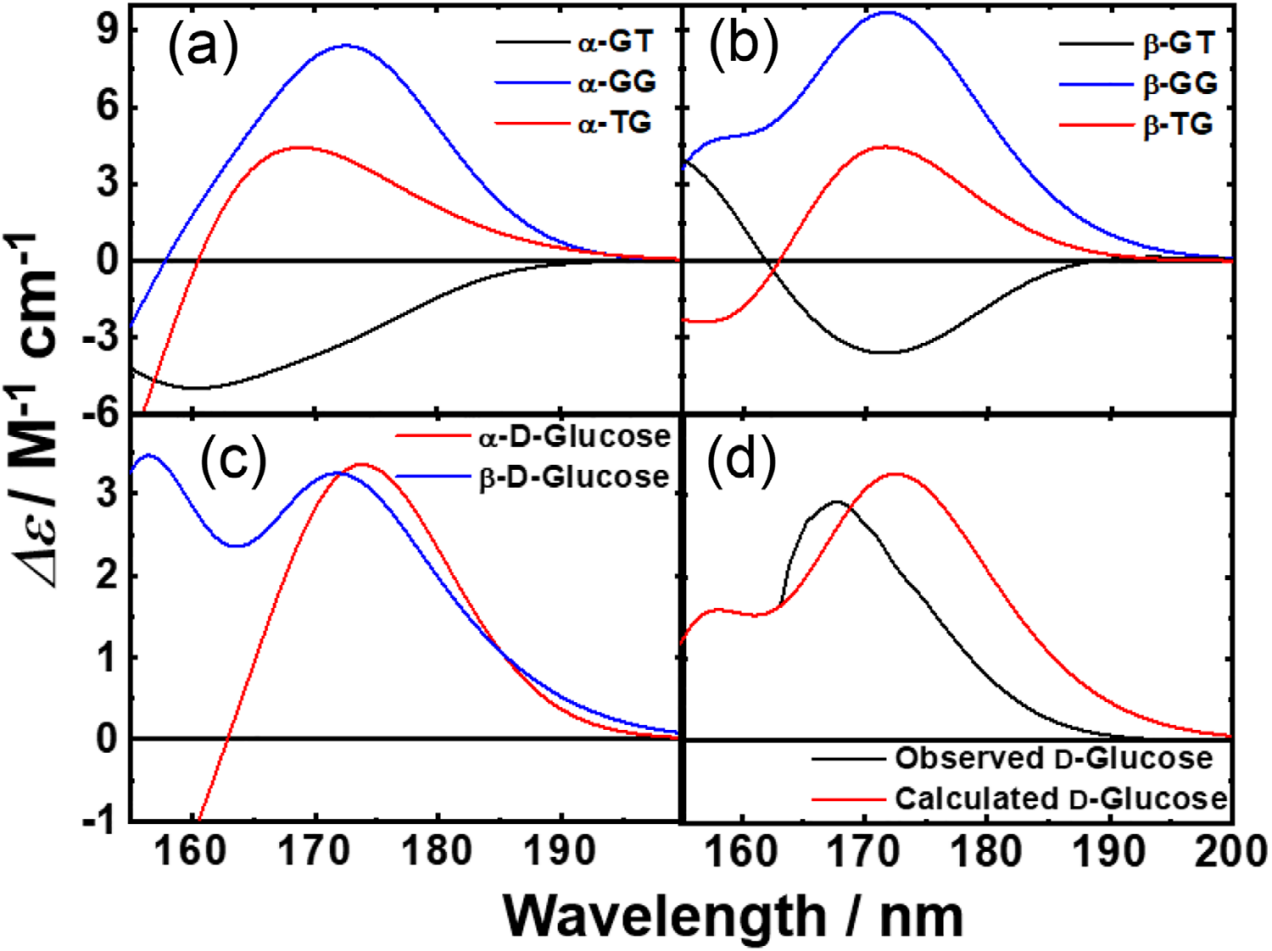
Theoretical CD spectra of d‐Glucose. (a) GT, GG, and TG rotamers of α‐d‐Glucose. (b) GT, GG, and TG rotamers of β‐d‐Glucose. (c) α‐d‐Glucose and β‐d‐Glucose spectra estimated based on a linear combination of the GT, GG, and TG spectra using their population ratios. (d) Experimentally observed and theoretically calculated spectra of d‐Glucose estimated as a linear combination of the α‐d‐Glucose and β‐d‐Glucose spectra using their population ratios. Adapted from Matsuo et al. (2020) [87].

The calculated CD spectra of the six conformers of d‐Glc (α‐GT, α‐GG, α‐TG, β‐GT, β‐GG, and β‐TG) are shown in Figure 13a,b. Among them, the GT and GG conformers are the most distinctive, producing opposite signals around 170 nm, negative for GT and positive for GG, in both α‐ and β‐anomers. To reconstruct the spectra of α‐d‐Glc and β‐d‐Glc, the individual contributions of their three main conformers (α‐GT, α‐GG, α‐TG and β‐GT, β‐GG, β‐TG, respectively) were combined according to their relative populations (Figure 13c) [92]. The resulting spectra featured a band near 170 nm, slightly stronger for α‐d‐Glc, and a crossover point at ∼185 nm. These theoretical spectra were in close agreement with the experimental data.

The overall spectrum of d‐Glc, obtained by summing the weighted contributions of all six conformers, is presented in Figure 13d. Comparison with the experimental spectrum revealed strong consistency: both showed peak of similar intensity at ∼170 nm and nearly identical spectral shapes across the 200–163 nm region. These results demonstrate that the computational approach accurately reproduces the experimental spectrum of d‐Glc and capture key solution‐state properties, including conformational dynamics, intramolecular interactions, and hydration behavior of d‐Glc.

**FIGURE 14:**
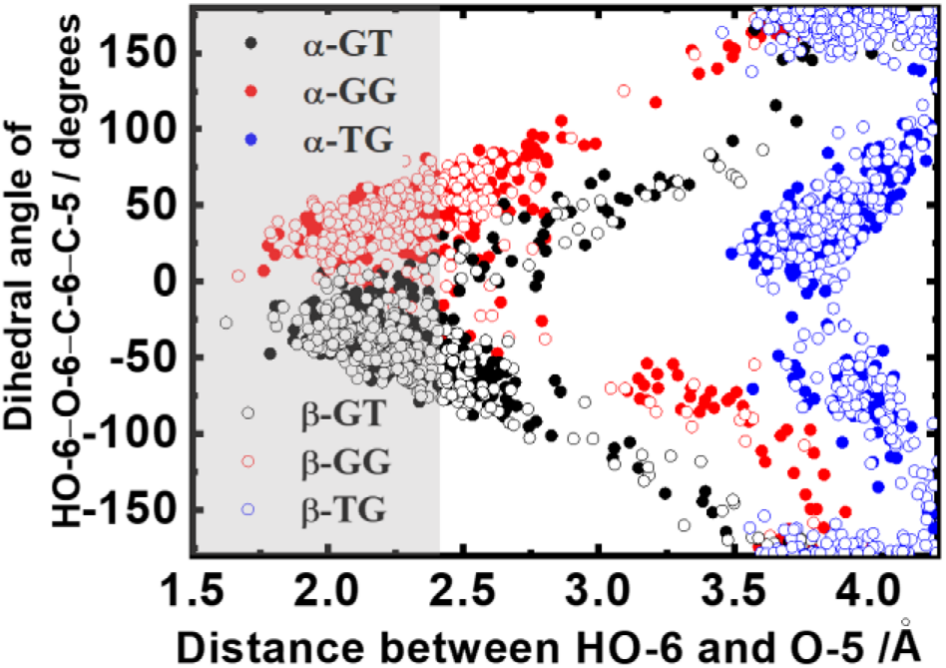
Plots of dihedral angles (HO‐6–O‐6–C‐6–O‐5) versus the distance between HO‐6 and O‐5 atoms in α‐GT, α‐GG, α‐TG, β‐GT, α‐GG, and α‐TG rotamers. The grey area indicates interatomic distance within 2.4 Å. Adapted from Matsuo et al. (2020) [87].

The structural dynamics and hydrogen‐bonding patterns of the six conformers were investigated using MD simulations. Figure 14 presents the relationship between the dihedral angle HO‐6–O‐6–C‐6–C‐5 and the HO‐6···O‐5 distance for the GT, GG, and TG rotamers of α‐d‐Glc. In the GT rotamer, the dihedral angle was near −50°, while in the GG rotamer it converged around 50°, both occurring at an interatomic distance of ∼2.4 Å, consistent with hydrogen bond formation between HO‐6and the ring oxygen O‐5 in both rotamers. By contrast, the TG rotamer did not show a consistent dihedral angle at the distance of 2.4 Å, indicating the absence of a HO‐6···O‐5 hydrogen bond in this rotamer. Instead, the TG rotamer exhibited alternative interactions: the HO‐4–O‐4–C‐4–C‐3 dihedral angle converged to ∼50° with an HO‐6···O‐4 distance of ∼2.4 Å, and to ∼ −150° with an O‐6···HO‐4 distance of ∼2.4 Å [87]. These results suggest that two distinct hydrogen bonds, O‐6–HO‐4 and O‐4–HO‐6, are possible in the TG rotamer.

To assess the stability of intramolecular hydrogen bonds involving the hydroxyl group at C‐6, the total retention time of these bonds was calculated by counting the number of frames within an interatomic distance of 2.4 Å. Over a 20 ns simulation, the hydrogen bond lifetimes were 11.2 ns for α‐GT, 13.3 ns for α‐GG, 16.7 ns for α‐TG, 12.7 ns for β‐GT, 13.4 ns for β‐GG, and 16.4 ns for β‐TG. These values indicate that the TG rotamers maintain hydrogen bonding at C‐6 more persistently than the GT and GG rotamers.

The MD simulations also provided insight into hydration levels, estimated from the number of surrounding water molecules forming hydrogen bonds with the solute (defined by an H–OA distance ≤ 2.4 Å and an OD–H–OA angle >120°, where OD and OA are donor and acceptor oxygen atoms). The C‐6 hydroxyl groups in α‐TG and β‐TG interacted with fewer water molecules (≈1.5) compared to those in the α‐GT, β‐GT, α‐GG, and β‐GG rotamers (1.7–2.1). Notably, the longer hydrogen bond retention times in the α‐TG and β‐TG rotamers (16.1–16.7 ns) coincided with lower hydration numbers relative to the other rotamers (11.2–13.4 ns).

**FIGURE 15:**
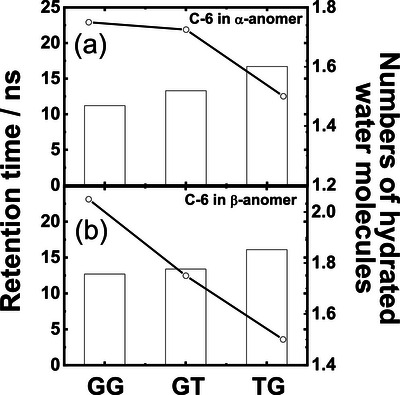
Retention time (histogram) and number of hydrating water molecules (circles and lines) associated with the hydroxyl groups at C‐6 in the (a) α‐anomer and (b) β‐anomer. Adapted from Matsuo et al. (2020) [87].

Figure 15 illustrates the relationship of hydrogen bond retention times and hydration numbers for the C‐6 hydroxyl group in both α‐ and β‐anomers. The analysis revealed a strong inverse correlation between hydrogen bond persistence and hydration, with correlation coefficients of −0.99 for the α‐anomer and −0.95 for the β‐anomer. Comparable trends were also observed for the hydroxyl groups at positions C‐4, C‐3, C‐2, and C‐1.

Thus, stronger intramolecular interactions are associated with reduced hydration around chromophores, suggesting that hydration is a key factor for stabilizing rotamer and anomer structures. This analysis reveals strong relationships among structural dynamics, intramolecular interactions, and degree of hydration in d‐Glc.

### 3.6 Oligosaccharide CD Spectra

**FIGURE 16:**
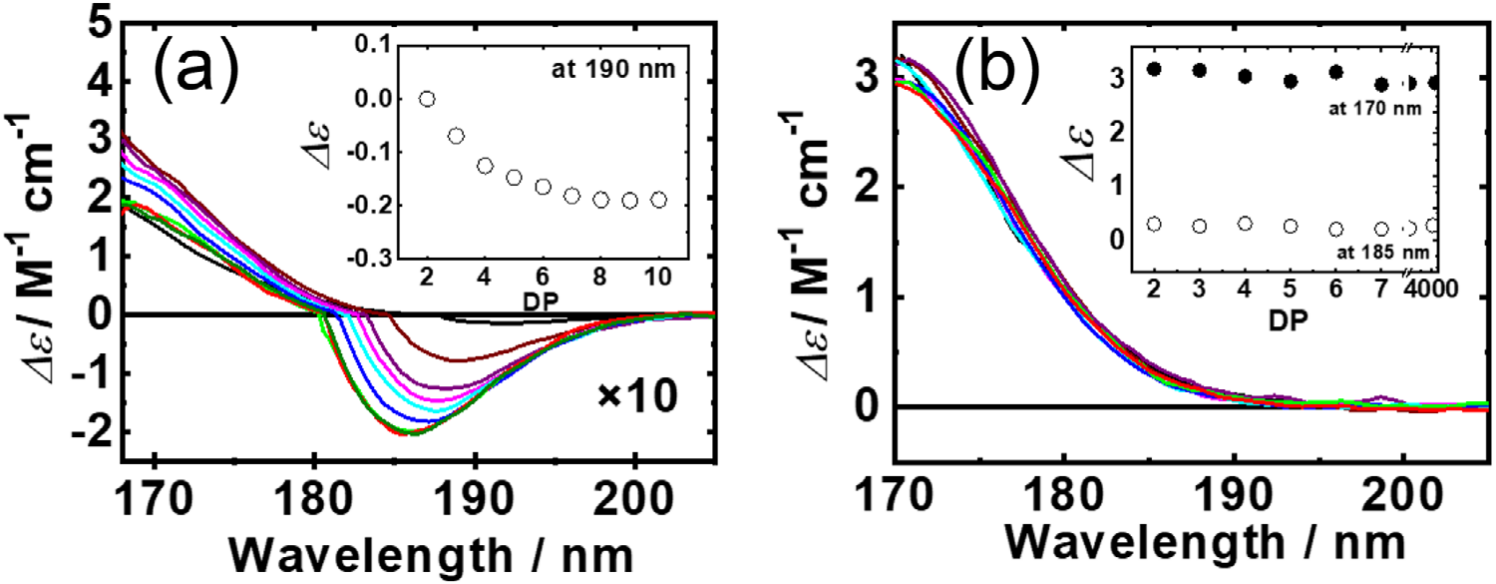
VUVCD spectra of oligosaccharides in aqueous solution at 25°C. (a) Malto‐oligosaccharide series (maltose, black; maltotriose, brown; maltotetraose, violet; maltopentaose, pink; maltohexaose, sky blue; maltoheptaose, blue; maltooctaose, green; maltononaose, red; and maltodecaose, dark green). The intensities of CD in the negative region are shown as 10‐fold magnified spectra. (b) Isomalto‐oligosaccharide series (isomaltose, black; isomaltotriose, brown; isomaltotetraose, violet; isomaltopentaose, pink; isomaltohexaose, sky blue; and isomaltoheptaose, blue) and dextran (DP = 31, green; and DP = 4000, red). These sample solutions were prepared at concentrations ranging from 1.0% to 8.0% (w/v).

Maltose, a disaccharide consisting of two d‐Glc residues linked by an α‐(1→4)‐glycosidic bond, was used for analyzing the relationships between conformations and CD spectra of malto‐oligosaccharides. The VUVCD spectra of nine malto‐oligosaccharides (degree of polymerization, DP = 2–10) in the 168°–200 nm region, are shown in Figure 16a. All spectra shared similar overall features; however, with increasing chain length, the negative CD band near 190 nm gradually shifted to shorter wavelengths and its intensity increased. The inset of Figure 16a illustrates the relationship between CD intensity at 190 nm and DP, demonstrating an exponential decrease in intensity, which saturated around DP ≈ 8.

Isomaltose, composed of two d‐Glc residues linked through an α‐(1→6)‐glycosidic bond, serves as the repeating unit of dextran. Figure 16b presents the VUVCD spectra of five isomalto‐oligosaccharides (DP = 2–6) and two dextran polysaccharides (DP = 31 and 4000) in the 170–260 nm range. The spectral profiles were similar across all saccharides, and the inset plot (Figure 16b) of CD intensity at ∼170 and ∼185 nm revealed no dependence on chain length. This suggests that, unlike the malto‐oligosaccharides, the isomalto‐oligosaccharide series does not display chain‐length–dependent variations in CD features.

The crystal structures of disaccharides were determined by x‐ray crystallography [93], showing that maltose formed intramolecular hydrogen bonds between HO’‐3…O‐2 (between two d‐Glc residues), while isomaltose did not participate in such bonding. Hence, to understand why malto‐ and isomalto‐oligosaccharides exhibit different chain‐length–dependent spectral behaviors, it is necessary to consider how glycosidic torsion angles change as the chain grows. Sugiyama et al. found that the torsion angles of glycosidic linkages in malto‐oligosaccharides (DP = 2–7) differed slightly from those of longer oligomers. They further proposed that short amylose chains containing about 16–17 glucose units adopt nearly three complete turns of the helical coil, forming a helical structure with 6–7 glucose residues per turn [93]. Figure 16a shows that CD intensities above 180 nm decrease progressively from DP = 2 to DP = 7 and saturate at around DP = 7–8 of glucose units, which is consistent with the number of glucose residues per turn mentioned above. This observation suggests that intermolecular hydrogen bonding among glucose residues stabilizes the regular helical conformation [94]. In contrast, dextran is known to adopt flexible, random conformations [95]. This flexibility likely arises from the α‐ (1→6) linkage in isomaltose units, which prevents the formation of stabilizing intermolecular hydrogen bonds between glucose residues [96]. As a result, the isomalto‐oligosaccharide series does not show chain‐length–dependent CD changes.

Overall, these findings demonstrate that VUVCD spectroscopy is highly sensitive to glycosidic torsion angles, providing a powerful means of monitoring the emergence of ordered saccharide structures in solution.

### 3.7 Biopolymers (Extracellular Polysaccharides and Polyhydroxyalkanoates)

**FIGURE 17:**
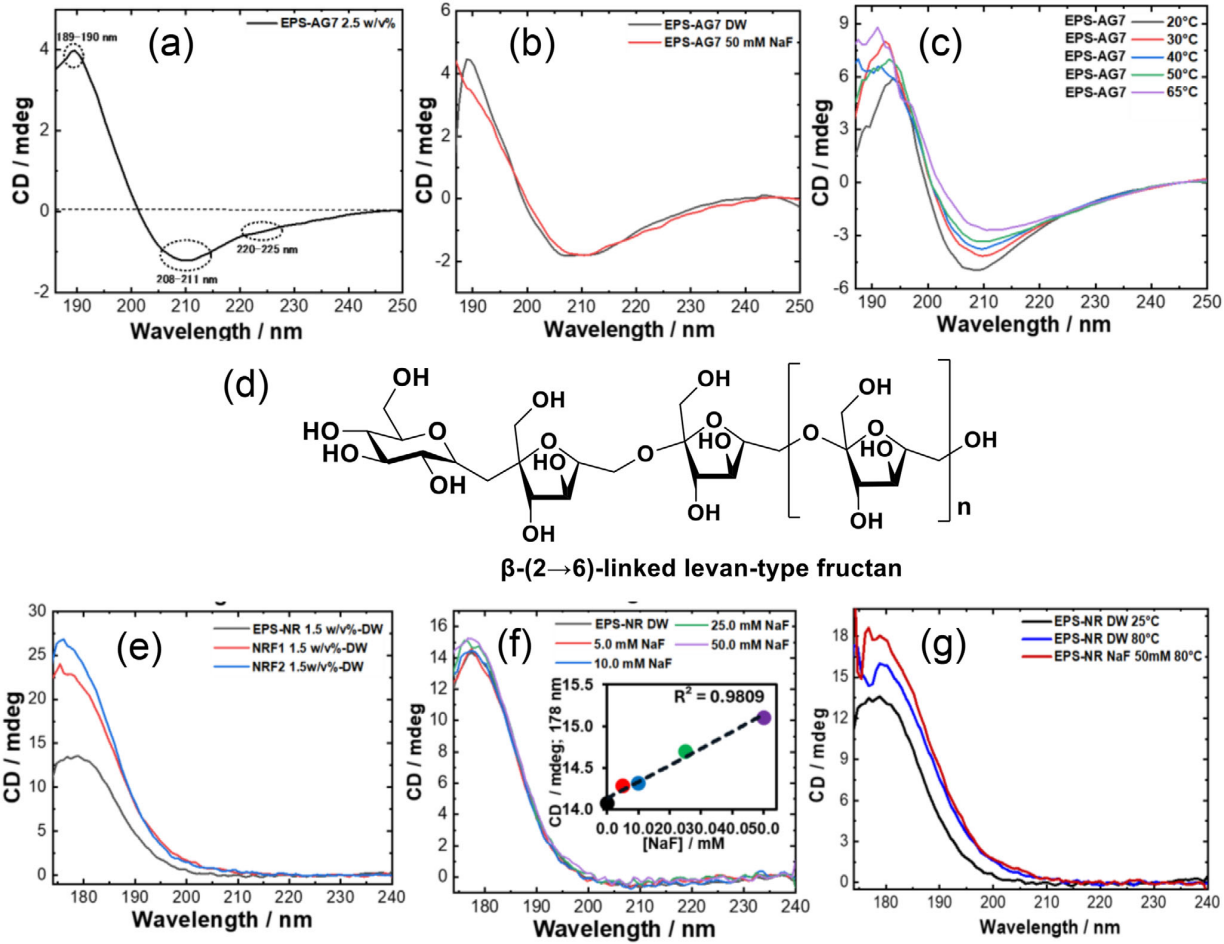
VUVCD analysis of EPSs. (a–c) EPS‐AG7 from *Aspergillus* sp. GAD7 showing (a) VUVCD spectrum (2.5 w/v% in DW, 25°C), (b) influence of high ionic strength at 50 mM NaF, and (c) temperature‐dependent spectra (20°C–65°C in DW). (d–f) EPS‐NR from *Bacillus paralicheniformis* ND2 showing (d) representative molecular structure of a levan‐type fructan composed of β‐(2→6)‐linked fructofuranosyl units, (e) CD spectra (1.5 w/v% in DW, 25°C), (f) influence of ionic strength in the presence of NaF (0–50 mmol/L), and (g) temperature‐dependent spectra at 25°C and 80°C in DW and NaF solution. Panels (a–c) adapted from Ibrahim et al. (2024). Panels (e–g) adapted with permission from El Halmouch et al. (2023) [21], [22].

VUVCD using SR light source has emerged as a powerful spectroscopic approach for investigating the structural and conformational properties of biopolymers, in particular polysaccharides. Compared to conventional CD, VUVCD provides an extended spectral window into the VUV region, thereby enhancing sensitivity to electronic transitions associated with specific molecular motifs such as glycosidic linkages, ring and ether oxygen atoms, and ordered backbone segments of polysaccharides [77, 87, 97]. The VUVCD measurements performed at HiSOR have been particularly instrumental in advancing such studies, allowing high‐resolution monitoring of subtle conformational changes induced by environmental factors such as concentration, ionic strength, temperature, and interactions with biomolecular partners. Recent work on EPS has demonstrated the value of VUVCD for uncovering structural signatures of complex carbohydrate assemblies. For example, an acidic, sulfated EPS (EPS‐AG7) derived from a marine *Aspergillus* sp. strain exhibited a triple‐helix–like arrangement, as revealed by negative Cotton effects at ∼222, ∼209 nm and positive Cotton effects at ∼189 nm [22]. These spectral features arise from cooperative electronic transitions along ordered polysaccharide backbone segments, stabilized by inter‐chain hydrogen bonding and electrostatic interactions involving sulfate substituents. Importantly, this helical arrangement proved stable across varying concentrations, and high ionic strength (Figure 17a,b). Only elevated temperatures produced slight reductions in ellipticity, consistent with the disruption of weak hydrogen bonding interactions (Figure 17c).

Complementary studies on levan‐type fructans produced by *Bacillus paralicheniformis* ND2 further highlight the versatility of VUVCD in EPS analysis [21]. In this case, VUVCD spectra revealed broad positive Cotton effects around 176–178 nm, which were attributed to electronic transitions associated with ether (C─O─C) linkages and ring oxygen atoms within the fructofuranosyl units (Figure 17d). Unlike the helical conformation observed in EPS‐AG7, the levan‐type fructans did not display well‐defined secondary structural bands, instead adopting unfolded (open‐chain) hydrated conformations stabilized primarily through intermolecular hydrogen bonding with water molecules, and ionic strength had little impact (Figure 17e,f). Temperature, however, induced minor conformational rearrangements, reflecting the sensitivity of EPS solvation dynamics (Figure 17g).

These studies underscore the unique ability of VUVCD instrumentation to dissect the structural and conformational analysis of EPS under physiologically relevant conditions by directly linking CD signatures to defined molecular motifs. The comparative insights between fungal and bacterial EPSs not only reveal the diversity of EPSs conformations, ranging from ordered triple helices to undefined hydrated structures, but also demonstrate how subtle environmental perturbations can dictate structural stability. These findings pave the way for broader applications of VUVCD in polymer science, where monitoring conformation is critical for linking molecular architecture to functional properties.

Importantly, comparable trends have been independently reported by multiple international research groups using conventional CD, induced CD (ICD), and complementary scattering techniques, underscoring the global relevance of CD‐based polysaccharide conformational analysis. Early foundational studies established CD, particularly Congo red–induced CD (ICD), as a sensitive probe for ordered polysaccharide helices. Ogawa and Hatano demonstrated that bacterial (1→3)‐β‐d‐glucans (curdlan) undergo helix–coil transitions that generate characteristic ICD signals upon Congo red binding, with these spectroscopic changes corroborated by x‐ray diffraction and NMR analyses [98]. A study by Du et al. further linked polysaccharide helicity to biological function. An EPS from *Schizophyllum commune*, composed of a β‐(1→3)‐glucan backbone with side chains, exhibited a positive Cotton effect near 195 nm in CD spectra of its Congo red complex, consistent with an ordered helical structure and correlated with pronounced anti‐inflammatory activity [99].

Advances in nanoscale and chemically modified polysaccharides have revealed additional structural diversity. Formylated yeast β‐glucan was shown to self‐assemble into chiral helical nanostructures, with strong ICD signals arising only upon complexation with Congo red. Split Cotton effects (positive at ∼500 nm and negative at ∼560 nm) indicated right‐handed helicity, while enhanced bands near ∼600 nm for nanorod dimers were consistent with single‐helix conformations, demonstrating that chemical modification can shift β‐glucans from native triple helices toward single‐helical architectures [100]. Conformational sensitivity captured by CD has also been linked directly to biological performance. Acid heteropolysaccharides isolated from steamed ginseng (WSGP‐S3 and WSGP‐G3) exhibited distinct CD signatures reflecting different chain organizations. The helix‐like WSGP‐G3 showed a positive Cotton effect at ∼197.6 nm and a negative band at ∼224.7 nm, associated with α‐anomer dominance and *n*→π* transitions of carboxyl groups. Despite its higher structural order, WSGP‐G3 displayed weaker biological efficacy than the non‐helical, branched WSGP‐S3 [101]. The integration of CD with complementary structural techniques has further enhanced mechanistic insight. Combined CD and SAXS studies on xanthan revealed distinct kinetic sensitivities to local and global structural rearrangements during helix–coil transitions. CD detected rapid changes in local chiral environments, while SAXS captured slower backbone reorganization, providing evidence for a metastable loose double‐helical intermediate [102]. More recently, CD spectroscopy has been widely applied to complex multicomponent systems. Zhang et al. showed that different polysaccharides distinctly regulate the secondary structure and functional properties of soybean protein isolate–quercetin complexes. Far‐UV CD (190–250 nm) revealed significant reductions in α‐helix and β‐sheet content accompanied by increased β‐turns and random coils, with sodium alginate inducing a stronger disordering effect than dextran. These CD‐detected conformational changes directly correlated with enhanced emulsifying, foaming, and antioxidant properties [103]. Extraction‐dependent conformational modulation has also been demonstrated in food‐relevant polysaccharides. CD studies on *Pleurotus ostreatus* polysaccharides showed that aqueous two‐phase system extraction produced a negative Cotton effect near ∼212 nm, whereas hot‐water extraction yielded a stronger band near ∼223 nm. The results indicate similar backbone conformations, with increased ellipticity arising from partial disruption of weak hydrogen‐bonded assemblies rather than changes in the fundamental chain architecture [104].

**FIGURE 18:**
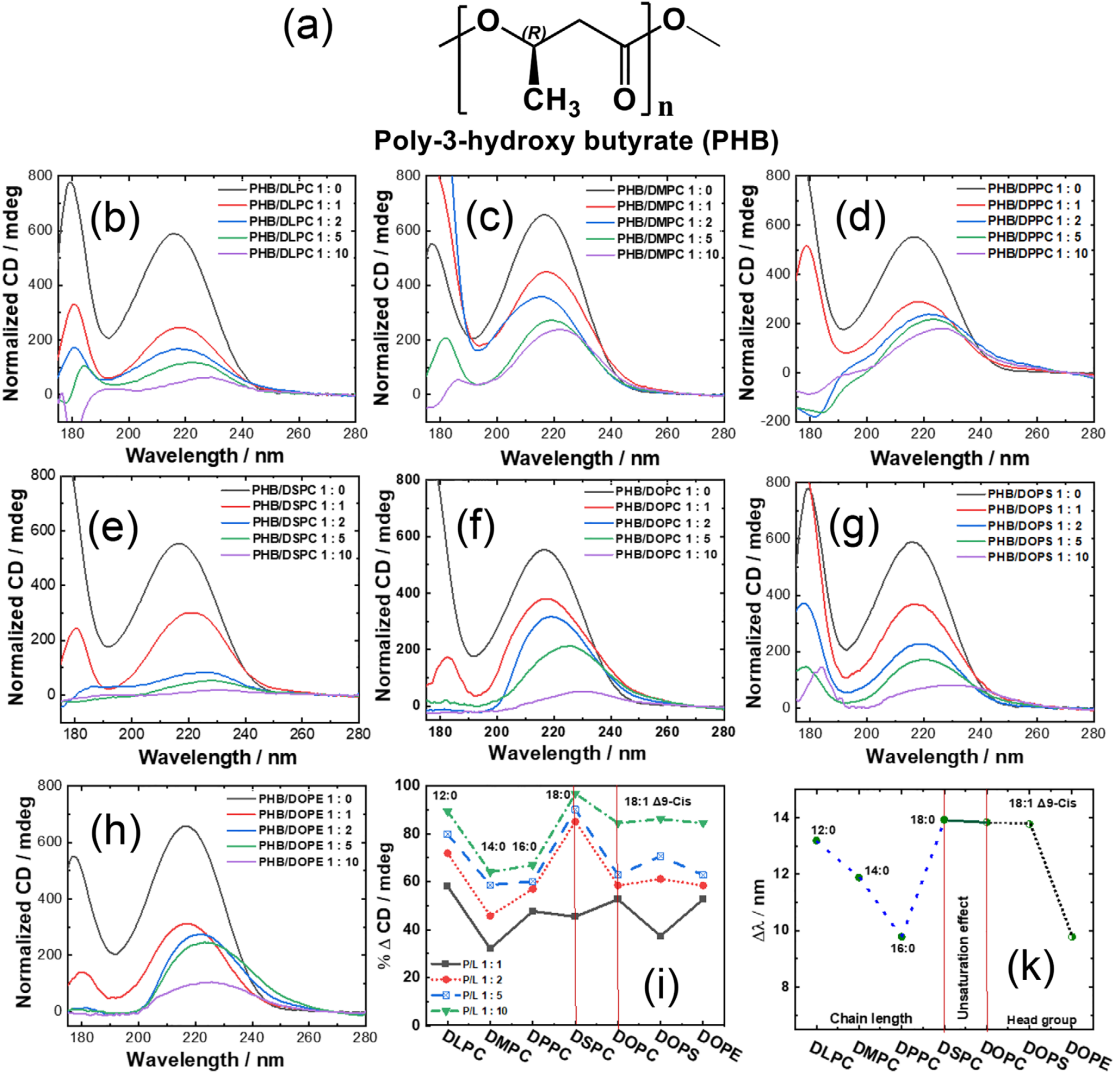
(a) Representative chemical structure of poly‐(R)‐3‐hydroxybutyrate (PHB), highlighting the chiral backbone and ester carbonyl groups. (b–h) Normalized CD spectra of PHB–lipid films at different lipid‐to‐polymer (L/P) weight ratios: (b) PHB/DLPC, (c) PHB/DMPC, (d) PHB/DPPC, (e) PHB/DSPC, (f) PHB/DOPC, (g) PHB/DOPS, and (h) PHB/DOPE. Pure PHB films were prepared with PHB loadings of 0.15, 0.30, 0.40, and 1.00 mg per film, obtained by depositing 40 µL of a PHB solution in chloroform onto CaF2 cells. Lipid–PHB composite films were prepared at L/P weight ratios of 1:1, 2:1, 5:1, and 10:1 (w/w). Panels (i) and (k) summarize the influence of L/P ratio, lipid chain length, degree of unsaturation, and head group chemistry on the degree of interaction (ΔCD; i) and the level of PHB structuration (Δ*λ*; k), respectively. Adapted from Ibrahim et al. (2024) [24].

Beyond polysaccharides, VUVCD has also been applied to probe the conformation and intermolecular interactions of PHAs, a class of biodegradable polyesters with growing biomedical and industrial relevance. Owing to their ester‐rich backbone, PHAs exhibit characteristic VUVCD signals arising predominately from *n*→π* electronic transitions of ester carbonyl groups [8]. Recent studies have reported, for the first time, detailed structural assessments of poly‐3‐hydroxybutyrate (PHB), the most common PHA, and its interactions with lipid membranes (Figure 18a) [23, 24]. The CD spectra of PHB films revealed a broad positive Cotton effect centered at ∼217 nm, corresponding to the *n*→π* transition of the ester groups and backbone packing within PHB. This band served as a diagnostic marker for monitoring conformational changes upon interaction with lipid bilayers. Mixing PHB with different lipid membranes, including phosphatidylcholines (DMPC, DOPC, DLPC, DPPC, and DSPC), phosphatidylserine (DOPS), and phosphatidylethanolamine (DOPE), resulted in significant reductions in CD intensity and red shifts of the Cotton band (Figure 18b–h). These changes reflected strong lipid–polymer interactions, driven primarily by hydrophobic forces, with additional electrostatic contributions depending on the lipid head group. Among the tested lipids, DMPC (14:0) exhibited the strongest effect at low lipid‐to‐PHB ratios (1:1), inducing up to ∼82% signal reduction and a red shift of ∼9 nm, likely due to its short, saturated chains enhancing flexibility and hydrophobic contacts with PHB. Conversely, DOPS, bearing a negatively charged serine headgroup, showed weaker interactions and an early saturation effect, highlighting the influence of electrostatic repulsion in modulating PHB structuration. More extensive analyses further revealed that lipid chain length and flexibility were the dominant factors governing interaction strength. Longer saturated chains, as in DSPC (18:0), promoted stronger hydrophobic associations at high lipid:polymer ratios (10:1), yielding the largest bathochromic shifts (>13 nm). By contrast, the degree of unsaturation (e.g., DOPC vs DSPC) and the chemistry of the polar head group (PC, PS, and PE) exerted comparatively minor effects on CD intensity, although head group hydrophobicity could modulate conformational changes (Figure 18i,k). These findings provide the first spectroscopic evidence (e.g., VUVCD) that lipid chemistry can substantially modulate the conformation of PHB, potentially influencing its biocompatibility and functional integration into lipid‐rich biological environments. Thus, the observed CD changes can be directly attributed to the reorganization of ester‐rich backbone segments in response to different lipid environments. The application of VUVCD thus extends beyond natural polysaccharides to microbial‐derived biopolymers. By resolving subtle conformational signatures and environmental sensitivities, VUVCD enables deeper mechanistic understanding of how PHAs interact with biological membranes, an insight of direct relevance for biomedical materials, drug delivery systems, and sustainable bioplastics.

## 4 Comparisons of VUVCD With Other Structural Biology Techniques

Structural analysis of biomolecules relies on several powerful techniques with atomic‐level resolution, including x‐ray crystallography, cryo‐electron microscopy (cryo‐EM), and NMR spectroscopy, all of which are regarded as essential tools in structural biology. Nevertheless, x‐ray crystallography requires the formation of well‐ordered crystals and is therefore not well suited for observing dynamic structural changes. Cryo‐EM, while not requiring crystallization, is primarily applicable to large biomolecules or symmetric complexes with molecular weights on the order of 100–1000 kDa, and remains challenging for the analysis of small molecules or low‐molecular–weight proteins. NMR spectroscopy, like VUVCD, is applicable to samples in solution; however, it is typically limited to relatively small biomolecules below approximately 20–30 kDa. Although methodological advances have extended its applicability to systems approaching 100 kDa, [105] NMR may also be subject to certain intrinsic limitations, such as relatively low sensitivity, the requirement for relatively large amounts of sample, and the need for isotopic labeling and solvent substitution.

In contrast, VUVCD spectroscopy does not require crystallization and enables measurements in solution under conditions close to physiological environments, across a wide range of solvent conditions. Moreover, VUVCD is applicable to a broad variety of biomolecules, including macromolecular complexes, without strict limitations on molecular weight. However, the structural information obtained from VUVCD is generally limited to the molecular level, such as secondary structures of proteins, isomeric structures of saccharides, and backbone conformations of biopolymers. In addition, because strong absorption by additives often occurs in the VUV region, careful optimization of experimental parameters, including optical path length, sample concentration, and solvent composition, is required. Furthermore, when multiple molecular species or structural states coexist in a sample, the observed CD spectra represent ensemble averages, which can complicate data interpretation.

Meanwhile, significant progress has been made in VUVCD spectral analysis through the incorporation of advanced computational approaches, such as MD simulations and bioinformatics. These developments have enabled the extraction of structural information that was previously difficult to obtain, including 3D structural features of proteins based on CD theory [106] and conformational analyses of saccharide isomers existing in complex equilibria in solution [87]. Furthermore, as mentioned above, combining CD with other spectroscopic techniques, such as LD, has contributed to obtaining structural information that was previously unavailable.

VUVCD might be positioned as a technique that complements above essential structural analysis methods, but its range of application to biomolecules is extremely wide. Continued development through integration with cutting‐edge computational science and other experimental techniques would realize the acquisition of further unexplored structural information. The strength and depth of VUVCD spectroscopy is expected to play an increasingly important role in advancing structural studies of biomolecules.

## 5 Summary and Outlook

CD is a widely used technique for studying the structures of biomolecules, and its effectiveness has been dramatically increased by the use of SR as a light source. In recent years, its applications have further expanded through integration with computational approaches such as bioinformatics and experimentally based modeling, as well as with complementary experimental techniques, thereby enhancing its analytical capability. In this review article, we introduced VUVCD spectroscopy using SR at HiSOR, Hiroshima University, highlighting recent advancements in CD instrumentation and its applications to structural studies of proteins, saccharides, and biopolymers. As a joint‐usage and research center, HiSOR actively collaborates with domestic and international researchers, and the aforementioned measurement systems and analytical methods have significantly contributed to various research fields, including structural studies of proteins involved in toxin–antitoxin systems related to carcinogenesis [107], proteins associated with DNA damage repair under environmental stresses such as radiation [108], rheological studies of polysaccharides [102], research on the biological effects of homochirality loss, [109] and structural studies of chiral carbon nanotubes [110].

At present, VUVCD measurements are mainly conducted for solution samples. When applied to tiny and assembled samples such as amyloid fibrils, droplet samples, and hydrogels, analysis becomes challenging due to artifacts such as strong light scattering, orientation effects, and ensemble‐averaged signals. These limitations would potentially be addressed through advances in beamline design and VUVCD instrumentation, including the development of advanced optical systems employing multiple photo‐elastic modulators and Schwarzschild‐type focusing mirrors, [19, 111] as well as optimization for each specific measurement condition. In parallel, structural studies of single‐ and double‐stranded DNA and RNA using VUVCD combined with LD are steadily progressing, [18, 27] and further advances are expected in elucidating the relationship between molecular chirality and biological function of nucleic acids. Further, the measurements and spectral analyses of complex mixtures also remain a major challenge; however, recent developments in hyphenated approaches, such as the integration of liquid chromatography with VUVCD—are promising, as this technique may enable simultaneous component separation and acquisition of CD information. Because VUVCD is also capable of detecting chirality in inorganic materials, such technological progress may further allow its application to nanostructures, including multicomponent nanofibers, for evaluating their handedness.

Thus, continued improvements in optical and measurement systems and analytical methodologies are expected to further broaden the versatility of SR‐based VUVCD applications even more in the future.

## Conflicts of Interest

The authors declare no conflicts of interest.
